# SMAD2 *S*-palmitoylation promotes its linker region phosphorylation and T_H_17 cell differentiation in a mouse model of multiple sclerosis

**DOI:** 10.1126/scisignal.adr2008

**Published:** 2025-05-27

**Authors:** Mingming Zhang, Tao Yu, Yinong Liu, Xuan Lu, Wenzhe Chen, Lixing Zhou, Yuejie Xu, Min Yang, Andrew D. Miller, Hening Lin

**Affiliations:** 1 Howard Hughes Medical Institute; Department of Chemistry and Chemical Biology, Cornell University, Ithaca, NY 14853, USA; 2 Department of Chemistry and Chemical Biology, Cornell University, Ithaca, NY 14853, USA; 3 The Center of Gerontology and Geriatrics/National Clinical Research Center for Geriatrics, West China Hospital, Sichuan University, Chengdu 610041, China; 4 Department of Gastroenterology, Drum Tower Hospital Affiliated to Nanjing Medical University, Nanjing 210008, China; 5 Cornell University College of Veterinary Medicine, Department of Population Medicine and Diagnostic Sciences, Section of Anatomic Pathology, Ithaca, NY 14853, USA; 6 Department of Molecular Biology and Genetics, Cornell University, Ithaca, NY 14853, USA

## Abstract

The transcriptional regulators SMAD2 and SMAD3 share the same primary signaling pathway in response to the cytokine TGF-β. However, whereas SMAD2 stimulates the differentiation of naive CD4^+^ T cells into proinflammatory T helper 17 (T_H_17) cells, SMAD3 stimulates the differentiation of anti-inflammatory regulatory T (T_reg_) cells. Here, we report a dynamic SMAD2-specific posttranslational modification important for T_H_17 cell differentiation. SMAD2, but not SMAD3, was reversibly *S*-palmitoylated at Cys^41^ and Cys^81^ by the palmitoyltransferase DHHC7 and depalmitoylated by the acyl protein thioesterase APT2. As a result, SMAD2 was recruited to intracellular membranes where its linker region was phosphorylated, leading to its interaction with the transcriptional regulator STAT3. Nuclear translocation of the SMAD2-STAT3 complex induced the expression of their target genes that promoted T_H_17 cell differentiation. Perturbation of SMAD2-STAT3 binding by inhibiting the palmitoylation-depalmitoylation cycle suppressed T_H_17 cell differentiation and reduced disease severity in mice with experimental autoimmune encephalomyelitis (EAE), a model of multiple sclerosis (MS). Thus, the *S*-palmitoylation–depalmitoylation cycle mediated by DHHC7 and APT2 specifically regulates SMAD2, providing insights into the functional differences between SMAD2 and SMAD3 and the distinct role of SMAD2 in T_H_17 cell differentiation. The findings further highlight DHHC7 and APT2 as potential therapeutic targets for the treatment of T_H_17 cell–mediated inflammatory diseases, including MS.

## INTRODUCTION

As a potent immunosuppressive cytokine, transforming growth factor–β (TGF-β) suppresses the maturation and functions of anti-inflammatory effector/memory immune cells by inducing the differentiation of naive CD4^+^ T cells into regulatory T (T_reg_) cells (1). However, TGFβ also promotes the differentiation of proinflammatory T helper 17 (T_H_17) cells from naive CD4^+^ T cells (2–4). Th17 cells are characterized by the expression of retinoic acid receptor–related orphan receptor gamma t (ROR-γt, which is encoded by *RORC*) and interleukin-17 (IL-17, encoded by *IL17A*) (5). Many immune-related diseases, including multiple sclerosis (MS), inflammatory bowel disease (IBD), and rheumatoid arthritis (RA), exhibit chronic inflammatory conditions with imbalanced ratios of Treg cells to Th17 cells, suggesting that T cell differentiation plays a key role in immune diseases (6–8). Due to the substantial influence of the TGF-β signaling pathway on T cell differentiation, understanding how the distinct functions of TGF-β are regulated will be useful for treating immune-related diseases.

Intracellular transduction of the TGF-β signal is initiated by the binding of TGFβ to TGFβ receptor II (TGFβRII) on the plasma membrane. Subsequently, TGFβRII phosphorylates and activates TGFβ receptor I (TGFβRI). Mothers against decapentaplegic homolog 2 and 3 (SMAD2 and SMAD3) are highly homologous TGFβ receptor–regulated SMADs (R-SMADs) and serve as the primary responders to TGFβ (9, 10). They can be recruited and phosphorylated by the TGFβR at two serine residues in the carboxy-terminus (C-terminus) (Ser^465^ and Ser^467^ for SMAD2 and Ser^423^ and Ser^425^ for SMAD3) (11–13). SMAD4, as the only known human co-SMAD, partners with R-SMADs to recruit these transcriptional co-regulators to the complex (14). Although SMAD2 and SMAD3 share the same upstream signaling pathway and many downstream partners, their actions and responses to TGFβ are highly context-dependent (4). Typically, SMAD2 promotes Th17 cell development, whereas SMAD3 promotes Treg cell differentiation (7, 12, 15), even though a full consensus has not yet been reached (16). A previous study showed that the phosphorylation of SMAD2 Ser^255^ by extracellular signal–regulated kinase (ERK) promotes its interaction with the transcriptional regulator signal transducer and activator of transcription 3 (STAT3) and that the SMAD2-STAT3 complex promotes Th17 cell differentiation, whereas SMAD3 that is not phosphorylated at the C terminus interacts with STAT3 to promote Treg cell differentiation and repress Th17 cell differentiation (7). This finding suggests that SMAD2 Ser^255^ phosphorylation can tune the balance between these cell types, but how this phosphorylation process is regulated is largely unknown, especially considering that the phosphorylatable residues are conserved between SMAD2 and SMAD3. Understanding the regulatory mechanisms involved may provide new therapeutic strategies to control Th17 cell and Treg cell differentiation and treat immune-related diseases (4).

Cysteine palmitoylation (*S*-palmitoylation) is an important posttranslational modification (PTM) of proteins that regulates protein-membrane associations and protein-protein interactions (17–21). *S*-palmitoylation is catalyzed by 23 palmitoyl transferases in humans, which are known as DHHCs due to their conserved Asp-His-His-Cys (DHHC) sequence motif and the identified zinc-finger domain, whereas it is reversed by acyl-protein thioesterases (APT1, APT2, and ABHD family members) (22–24). Although DHHCs act on various substrate proteins (8, 19, 25–27), their physiological function is less well understood than that of other PTM enzymes, such as protein kinases, and the therapeutic potential of targeting DHHCs in immune-related diseases still has not been well defined.

Here, we found that SMAD2, but not SMAD3, was subjected to reversible *S*-palmitoylation at Cys^41^ and Cys^81^, which was mediated by DHHC7 (encoded by *Zdhhc7*) and APT2 (encoded by *LYPLA2*). Furthermore, DHHC7-catalyzed palmitoylation and APT2-mediated depalmitoylation of SMAD2 promoted its phosphorylation at Ser^245^, Ser^250^, and Ser^255^ at intracellular membranes and its consequent association with STAT3, as well as nuclear translocation of the activated SMAD2-STAT3 complex. Through this mechanism, the palmitoylation-depalmitoylation cycle of SMAD2 promoted Th17 cell differentiation. In addition, this *S*-palmitoylation-regulated differentiation of Th17 cells contributed to the development of experimental autoimmune encephalomyelitis (EAE), a mouse model of multiple sclerosis. Perturbation of the SMAD2 palmitoylation-depalmitoylation cycle by genetically deleting *Zdhhc7* or *Lypla2* repressed Th17 cell differentiation and reduced disease severity in mice. Therefore, our study identifies a previously uncharacterized mechanism for the regulation of SMAD2 activation and Th17 cell differentiation, which suggests that targeting the DHHC7- and APT2-mediated *S*-palmitoylation–depalmitoylation cycle of SMAD2 could serve as a potential therapeutic approach to treat autoimmune diseases.

## RESULTS

### SMAD2 is *S*-palmitoylated by DHHC7

**Fig. 1.**
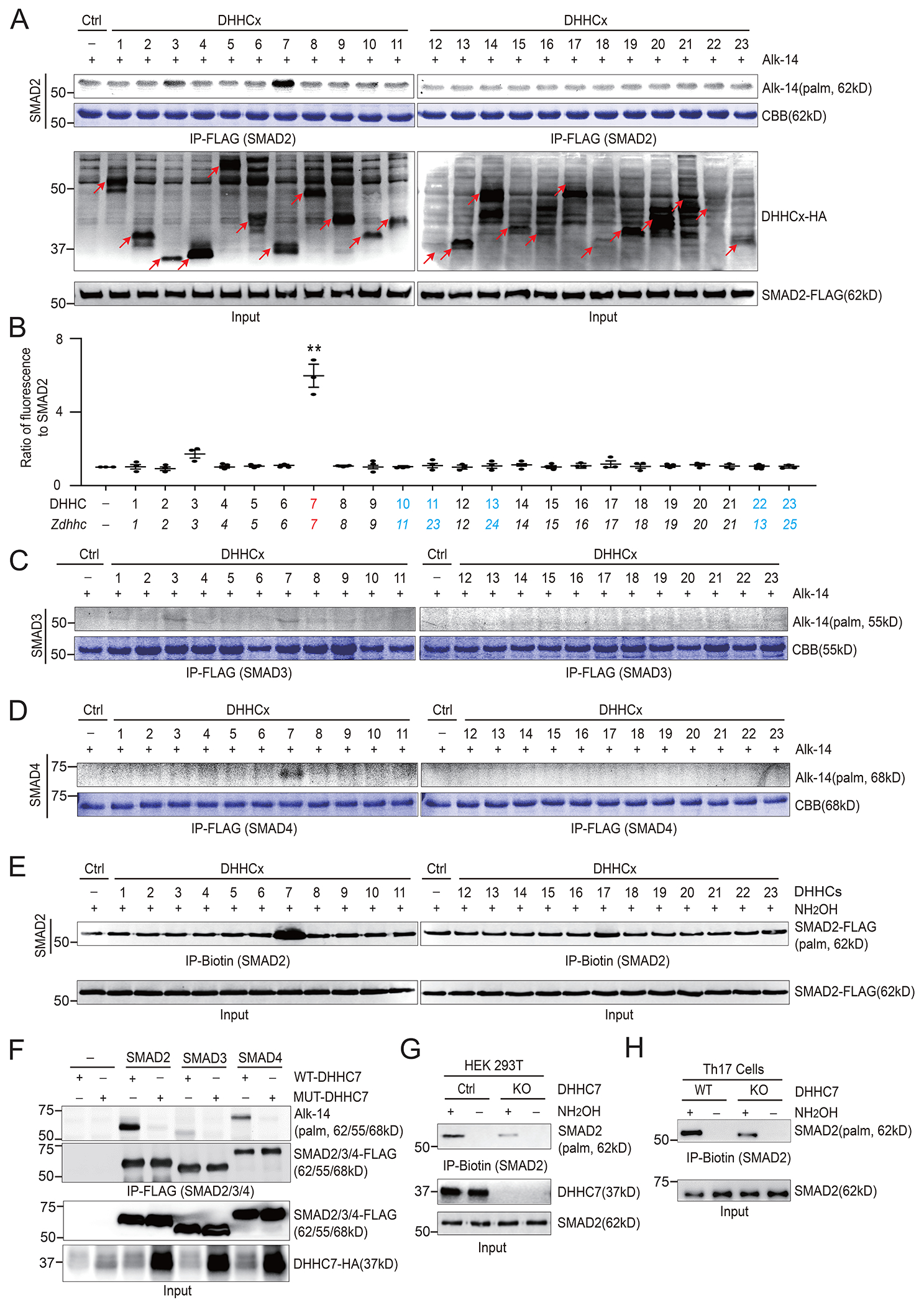
SMAD2 is palmitoylated by DHHC7. (**A**) Analysis of SMAD2 palmitoylation in HEK293T cells transfected with FLAG-SMAD2 and indicated HA-DHHC-encoding plasmids. The abundance of palmitoylated SMAD2 was determined by Alk14 metabolic labeling and in-gel fluorescence. Coomassie Brilliant Blue (CBB) staining was used to visualize total SMAD2 protein. (**B**) Quantification of relative amounts of palmitoylated SMAD2 in the experiments shown in (**A**). The amount of palmitoylated SMAD2 was normalized to the amount of total SMAD2 and to the amount in the control (with Alk14 but without DHHC overexpression), which was set to 1. All of the *DHHC* genes were from mouse, and the protein number corresponds to the gene number except for DHHC10 (Zdhhc11), DHHC11 (Zdhhc23), DHHC13 (Zdhhc24), DHHC22 (Zdhhc13), and DHHC24 (Zdhhc22) (18). (**C** and **D**) Analysis of the palmitoylation of SMAD3 (C) and SMAD4 (D) in HEK293T cells transfected with FLAG-SMAD3 or FLAG-SMAD4, respectively, and the indicated HA-DHHC-encoding plasmids. Palmitoylation of the indicated proteins was determined as described for (A). (**E**) Analysis of the palmitoylation of SMAD2 in HEK293T cells transfected with FLAG-SMAD2 and the indicated HA-DHHC–encoding plasmids. Palmitoylated SMAD2 was detected by acyl-biotin exchange (ABE) assay with NH_2_OH treatment. (**F**) Analysis of the palmitoylation of SMAD2, SMAD3, and SMAD4 in HEK293T cells transfected with the indicated FLAG-SMAD plasmids, together with plasmids encoding HA-tagged WT DHHC7 or a catalytically inactive mutant (MUT-DHHC7) . Palmitoylation was detected by Alk14 labeling and in-gel fluorescence. (**G**) Palmitoylation of endogenous SMAD2 in WT and DHHC7 KO HEK293T cells was detected with the ABE assay. (**H**) Palmitoylation of endogenous SMAD2 in WT and DHHC7 KO Th17 cells. Splenic CD4^+^ naïve T cells were isolated from WT and *Zdhhc7* KO mouse spleens and differentiated into Th17 cells in vitro. Palmitoylated SMAD2 was detected with the ABE assay. Quantified data for the experiments represented in (H) are shown in fig. S1D. Western blots are representative of three experiments. Quantified data are means ± SEM of three experiments. **P < 0.01.

Previous studies showed that inactive R-SMADs are recruited to the plasma membrane and intracellular membranes where TGFβR and ERK are located (28–30). We investigated whether *S*-palmitoylation could play a role in controlling the membrane association of R-SMADs, particularly SMAD2 and SMAD3, similar to the function of STAT3 *S*-palmitoylation, thus regulating the activation of SMAD2 and SMAD3 (collectively referred to as SMAD2/3) and T cell differentiation. We used metabolic labeling with Alk14, an alkyne-tagged palmitic acid analog, followed by TAMRA-azide conjugation through click chemistry and in-gel fluorescence to detect SMAD2/3 palmitoylation. We found that SMAD2 was labeled by Alk14 and that the extent of the labeling was significantly increased by DHHC7 expression (Fig. 1, A and B). Quantification of the palmitoylation signal showed that palmitoylated SMAD2 was six-fold more abundant in the presence of DHHC7 (Fig. 1B). Although DHHC3 also increased the abundance of palmitoylated SMAD2, the change was not statistically significant (Fig. 1B). In contrast, palmitoylated SMAD3 and SMAD4 were much lower in abundance, and the extent of palmitoylation remained low even when DHHC3 or DHHC7 was overexpressed (Fig. 1, C and D). Additionally, the acyl-biotin exchange (ABE) assay, another method to detect protein *S*-palmitoylation, was applied to visualize the *S*-palmitoylation signal of SMAD2 in HEK 293T cells expressing different DHHCs. Consistent with the Alk14 labeling data, only DHHC7 significantly increased the abundance of *S*-palmitoylated SMAD2 (Fig. 1E). Furthermore, a side-by-side comparison of SMAD2, SMAD3, and SMAD4 palmitoylation through Alk14 labeling with overexpression of wild-type (WT) DHHC7 or an inactive mutant DHHS7 supported the finding that SMAD2 was a much more effective substrate of DHHC7 (Fig. 1F and fig. S1A). Whereas SMAD3 and SMAD4 were also *S*-palmitoylated by DHHC7, the extent of their modification was much reduced, especially for SMAD3 (Fig. 1F and fig. S1A). Together, these data demonstrate that SMAD2 can be *S*-palmitoylated by the palmitoyl transferase DHHC7.

Next, to determine whether DHHC7 or DHHC3 served as the endogenous palmitoyl transferase for SMAD2, we generated DHHC7 and DHHC3 knockout (KO) HEK 293T cells and found that the abundance of *S*-palmitoylated endogenous SMAD2 was significantly reduced in DHHC7 KO cells (Fig. 1G) compared to that in wild-type (WT) cells, but not in DHHC3 KO cells (fig. S1B). In addition, the abundance of *S*-palmitoylated endogenous SMAD3 was unchanged in DHHC7 KO cells compared to that in WT cells (fig. S1C), suggesting that SMAD3 is not an endogenous substrate of DHHC7. Furthermore, the amount of *S*-palmitoylated SMAD2 was decreased in DHHC7-deficient primary mouse Th17 cells, for which SMAD2 has a dominant regulatory function in their differentiation (Fig. 1H and fig. S1D), further confirming that DHHC7 mediates SMAD2 *S*-palmitoylation under physiological conditions.

### Cys^41^ and Cys^81^ are the major palmitoylation sites of SMAD2

**Fig. 2.**
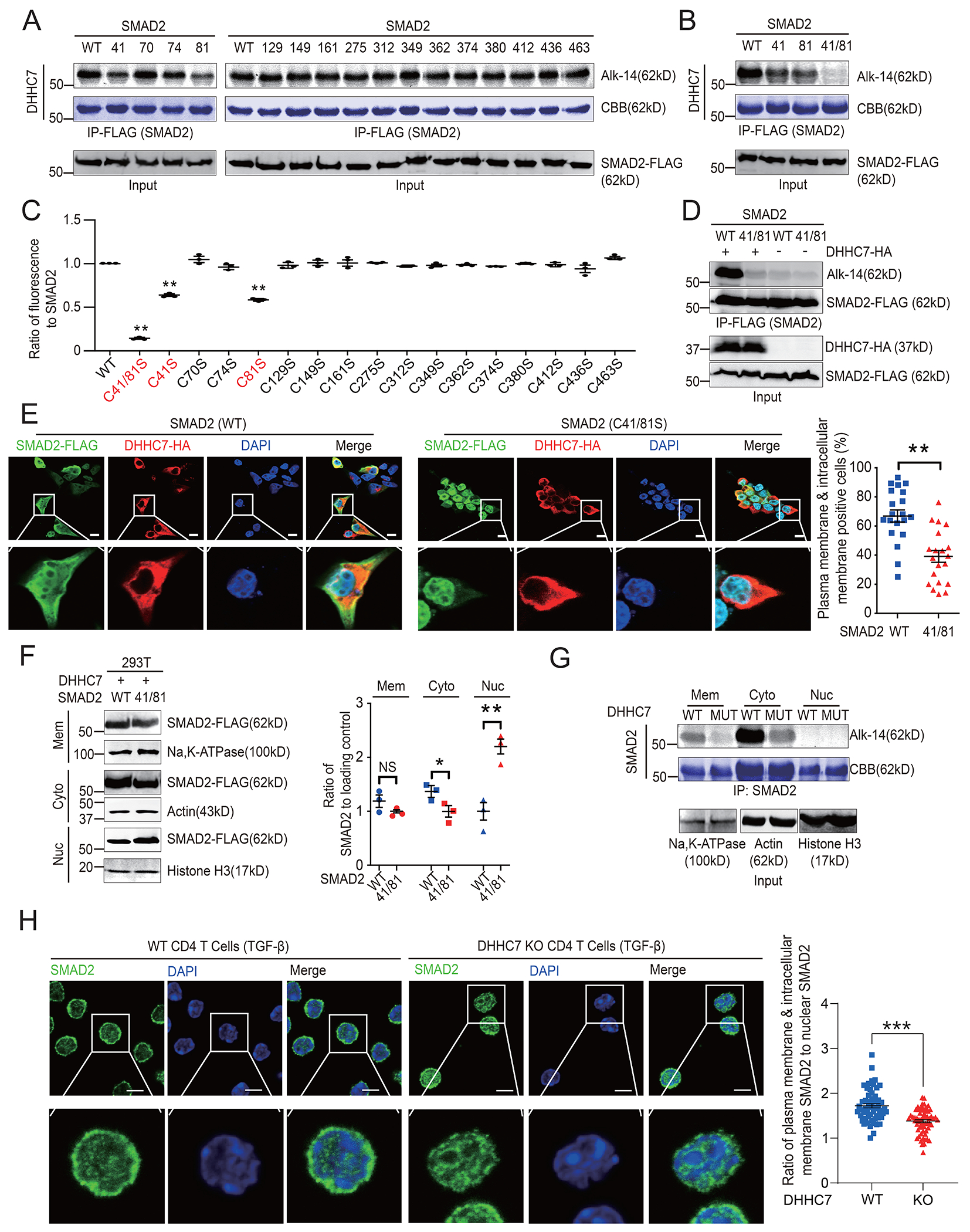
The palmitoylation of Cys^41^ and Cys^81^ promotes the membrane recruitment of SMAD2. (**A** and **B**) Analysis of the palmitoylation of SMAD2 in HEK293T cells transfected with HA-DHHC7 and plasmids encoding the indicated FLAG-SMAD2 cysteine site mutants. The palmitoylation of SMAD2 was determined by Alk14 labeling and in-gel fluorescence; CBB staining was used to visualize total SMAD2 protein. (**C**) Quantification of relative amounts of palmitoylated SMAD2 from the experiments shown (A) and (B). The relative amount of palmitoylated SMAD2 was normalized to the amount of total SMAD2 protein and the abundance in the WT SMAD2 sample, which was set to 1. (**D**) Analysis of the palmitoylation of SMAD2 in HEK293T cells transfected with or without HA-DHHC7. HEK293T cells were cotransfected with plasmids encoding FLAG-SMAD2 WT or Cys41Ser/Cys81Ser mutant (41/81), together with or without plasmid encoding HA-DHHC7, and then treated with Alk14. The amount of *S*-palmitoylated immunoprecipitated SMAD2 was visualized by in-gel fluorescence. (**E**) Left: The subcellular localization of FLAG-SMAD2 and HA-DHHC7 was analyzed by confocal imaging in HEK293T cells. Scale bars, 50 μm. Right: The percentage of DHHC7-positive cells in which SMAD2 translocated from the nucleus to the plasma membrane and endomembranes was quantified using Pearson’s correlation coefficient. Each point represents the percentage of positive cells in one random microscope field of view from three independent experiments. (**F**) Subcellular distribution of SMAD2 in the cytoplasmic (Cyto), plasma membrane (Mem), and nuclear (Nuc) fractions in DHHC7 KO HEK293T cells expressing WT or C41S/C81S mutant SMAD2. DHHC7 KO HEK293T cells were cotransfected with plasmids encoding HA-DHHC7 and the indicated FLAG-SMAD2 constructs (WT or Cys41/81Ser mutant), and subcellular fractionation was performed with cell lysates. Left: Equivalent amounts of fractions were then analyzed by Western blotting. Right: The relative amounts of SMAD2 in the indicated samples were quantified. (**G**) SMAD2 *S*-palmitoylation in different subcellular fractions of DHHC7 KO HEK293T cells ectopically expressing WT or a catalytically inactive mutant (MUT) DHHC7. The cells were transfected with plasmid encoding FLAG-SMAD2 and labeled with Alk14. Subcellular fractionation was performed and the amounts of palmitoylated immunoprecipitated SMAD2 in the cytoplasmic (Cyto), plasma membrane (Mem), and nuclear (Nuc) fractions were visualized by in-gel fluorescence. (**H**) Left: The subcellular localization of SMAD2 was analyzed by confocal imaging of WT and DHHC7 KO CD4^+^ T cells stimulated with TGFβ. Scale bars, 5 μm. Right: The ratio of the amounts of SMAD2 distributed in the plasma membrane and intracellular membranes to that in the nuclear fraction was quantified with Cell Profiler software. Each point represents one cell from three independent experiments. Quantified data are means ± SEM of three experiments. *P < 0.05, **P < 0.01, ***P < 0.001; NS, not significant.

To identify the potential palmitoylated cysteine(s) of SMAD2, we mutated each of the 15 cysteine residues in human SMAD2 to serines and used Alk14 labeling to determine the extent of *S*-palmitoylation of each mutant. The C41S and C81S mutations significantly reduced the amount of *S*-palmitoylated SMAD2, and the C41S/C81S double mutation abolished palmitoylation (Fig. 2, A to C). Furthermore, DHHC7 overexpression promoted the *S*-palmitoylation of WT SMAD2, but not of the C41S/C81S mutant (Fig. 2D), suggesting that DHHC7-catalyzed SMAD2 palmitoylation occurs mainly at these two cysteines.

### *S*-palmitoylation promotes the localization of SMAD2 to the plasma membrane and intracellular membranes

*S*-palmitoylation can target proteins to cellular membranes (19, 31, 32). SMAD2 must be recruited to the plasma membrane and intracellular membrane organelles (such as endosomes) to interact with TGFβR and ERK, respectively (12, 28, 33). Therefore, we studied the effect of *S*-palmitoylation on the membrane localization of SMAD2. Confocal imaging revealed that SMAD2 was localized in the nucleus, at the plasma membrane, and at intracellular membranes (Fig. 2E). The C41S/C81S mutant SMAD2 showed increased nuclear localization and decreased membrane localization compared to WT SMAD2 (Fig. 2E). To further confirm this finding, we fractioned total cell lysates into nuclear, plasma membrane, and cytoplasmic fractions (which includes intracellular membranes). Compared to WT SMAD2, the C41S/C81S mutant SMAD2 was more abundant in the nuclear fractions and less abundant in the cytoplasmic fractions (Fig. 2F), consistent with the results of our confocal imaging experiments. Moreover, DHHC7 expression increased the abundance of *S*-palmitoylated SMAD2 in the plasma membrane and cytoplasmic fractions, but not in the nuclear fraction (Fig. 2G), further supporting that palmitoylation promotes the plasma membrane and intracellular membrane localization of SMAD2. We also observed that SMAD2 was mainly localized at the plasma membrane and in the cytoplasm in TGF-β–stimulated WT CD4^+^ T cells, whereas SMAD2 showed increased nuclear localization in DHHC7-deficient cells (Fig. 2H), consistent with the earlier analysis of the localization of C41S/C81S SMAD2. Together, these findings indicate that DHHC7-catalyzed *S*-palmitoylation of SMAD2 at Cys^41^ and Cys^81^ promotes its localization at the plasma membrane and intracellular membranes and inhibits its nuclear distribution.

### DHHC7-dependent *S*-palmitoylation promotes the phosphorylation of SMAD2 at its linker region, but not in its C terminus

**Fig. 3.**
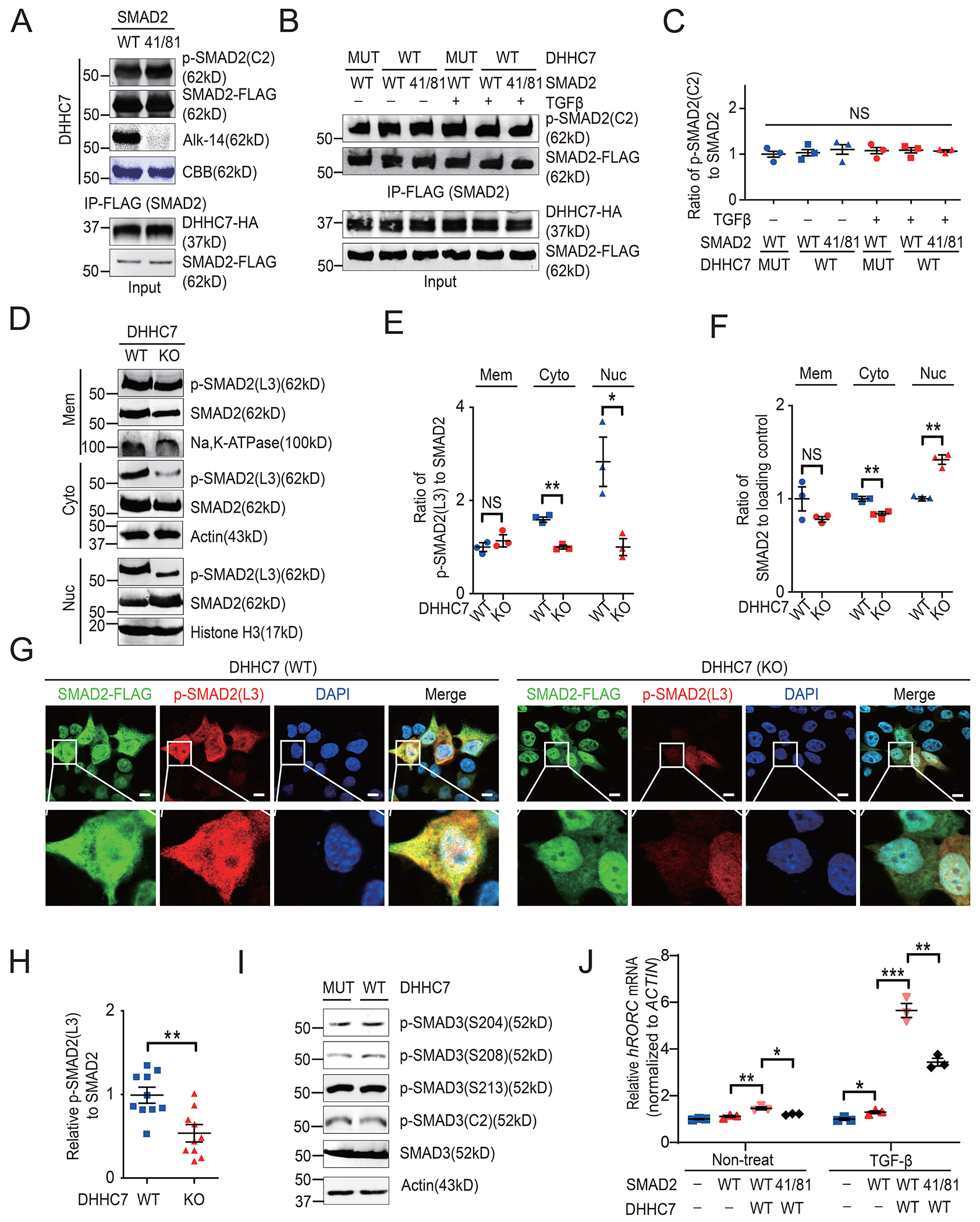
DHHC7-induced *S*-palmitoylation promotes phosphorylation of SMAD2 at the linker region, but not at the C terminus. (**A**) Analysis of the phosphorylation of SMAD2 at the C-terminal region [p-SMAD2(C2)] in HEK293T cells transfected with plasmids encoding HA-DHHC7 and FLAG-tagged WT or Cys41/81Ser mutant (41/81) SMAD2 was detected by Western blotting, and the palmitoylation of SMAD2 was determined by Alk14 labeling and in-gel fluorescence. (**B**) Phosphorylation of SMAD2 at the C-terminal region [p-SMAD2(C2)] in HEK293T cells cotransfected with plasmids encoding DHHC7-HA and FLAG-tagged WT or Cys41/81Ser mutant (41/81) SMAD2 and treated with TGFβ (3 ng/ml) for 6 hours. SMAD2 was pulled down with anti-FLAG resin and subjected to Western blotting analysis. (**C**) Quantification of relative amounts of p-SMAD2(C2) to that of total SMAD2 protein from the experiments shown in (B). (**D** to **F**) Distribution of SMAD2 and its phosphorylation at the linker region [p-SMAD2(L3)] in different subcellular fractions of WT and DHHC7 KO HEK293T cells. (D) Western blotting analysis of the indicated proteins in subcellular fractions from WT and DHHC7 KO cells. (E and F) Quantification of relative amounts of total SMAD2 and p-SMAD2(L3) in the indicated subcellular fractions. (**G**) The subcellular localization of SMAD2 and p-SMAD2(L3) in WT and DHHC7 KO HEK293T cells transfected with plasmid encoding FLAG-SMAD2 was analyzed by confocal imaging. Scale bars, 50 μm. (**H**) The relative amount of p-SMAD2(L3) in the experiments shown in (G) was quantified and normalized to that of total SMAD2, with the level in WT cells being set to 1. Each point represents one cell from three independent experiments. (**I**) The phosphorylation of SMAD3 at the linker region (Ser^204^/Ser^208^/Ser^213^) in HEK293T cells expressing WT or an inactive mutant of DHHC7 (MUT) was analyzed by Western blotting. (**J**) The relative amount of human *RORC* mRNA in HEK293T cells co-expressing WT or Cys41/81Ser mutant (41/81) SMAD2 with DHHC7 was determined by real-time PCR (Q-PCR) and was normalized to that of *ACTIN* mRNA. Quantified data are means ± SEM of three independent experiments. *P < 0.05, **P < 0.01.

The transcriptional functions of SMAD2 and SMAD3 are activated by phosphorylation at the C-terminal region (Ser^465^/Ser^467^ for SMAD2 and Ser^423^/Ser^425^ for SMAD3) (11–13). We previously found that DHHC7-induced *S*-palmitoylation could promote the phosphorylation and transcriptional activity of STAT3, another transcription factor that mediates Th17 cell differentiation (8). Therefore, we hypothesized that the *S*-palmitoylation of SMAD2 might similarly promote its C-terminal Ser^465^/Ser^467^ phosphorylation and activate its transcriptional function. Hence, we examined whether co-expression of DHHC7 with SMAD2 would increase the abundance of SMAD2 phosphorylated at Ser^465^/Ser^467^ [referred to as p-SMAD2(C2)] in HEK-293T cells; however, we unexpectedly found that it had no effect (fig. S2). Furthermore, SMAD2 and its *S*-palmitoylation-deficient C41S/C81S mutant resulted in similar amounts of p-SMAD2(C2) under basal conditions (Fig. 3A), as well as under conditions of DHHC7 expression and TGFβ stimulation (Fig. 3, B and C). Therefore, DHHC7-induced *S*-palmitoylation of SMAD2 did not affect its C-terminal phosphorylation at Ser^465^/Ser^467^.

SMAD2 and SMAD3 contain an N-terminal Mad homology-1 (MH1) domain, a linker region, and a C-terminal MH2 domain (7). Phosphorylation at the linker region residues Ser^245^/Ser^250^/Ser^255^ for SMAD2 and Ser^204^/Ser^208^/Ser^213^ for SMAD3 is important for their association with co-transcriptional factors and their transcriptional activity (34). Therefore, we investigated whether the phosphorylation of SMAD2 at the linker region [resulting in p-SMAD2(L3)] was regulated by SMAD2 palmitoylation. Unlike for phosphorylation at the C-terminal region, the abundance of p-SMAD2(L3) was significantly decreased in DHHC7 KO cells compared to that in WT cells. Loss of DHHC7 decreased p-SMAD2(L3) abundance in the cytoplasmic and nuclear fractions relative to that in WT cells (Fig. 3, D to F). Note that the ratio of p-SMAD2(L3) to SMAD2 abundance decreased even more in the nuclear fraction than in the cytoplasmic fraction (Fig. 3, D and E). Consistent with this, immunofluorescence data showed that SMAD2 was more abundant in the cytoplasm and that p-SMAD2(L3) was more abundant in WT cells than in DHHC7 KO cells (Fig. 3, G and H), further indicating that DHHC7-catalyzed SMAD2 *S*-palmitoylation promoted its phosphorylation at the linker region. In addition, although the sequence and function of SMAD3 are very similar to those of SMAD2, and both proteins share conserved phosphorylation sites in the linker domain (7), our data suggest that phosphorylation of the SMAD3 linker region, which was not *S*-palmitoylated (Fig. 1C and F, fig. S1A), was unaffected by DHHC7 (Fig. 3I), implying that regulation of linker region phosphorylation by DHHC7-mediated *S*-palmitoylation is specific for SMAD2. Consequently, SMAD2-mediated *RORC* expression was markedly enhanced by DHHC7 expression and significantly reduced in cells expressing the C41S/C81S mutant SMAD2 compared to that in cells expressing WT SMAD2 (Fig. 3J).

### APT2 depalmitoylates SMAD2 and regulates its linker region phosphorylation

**Fig. 4.**
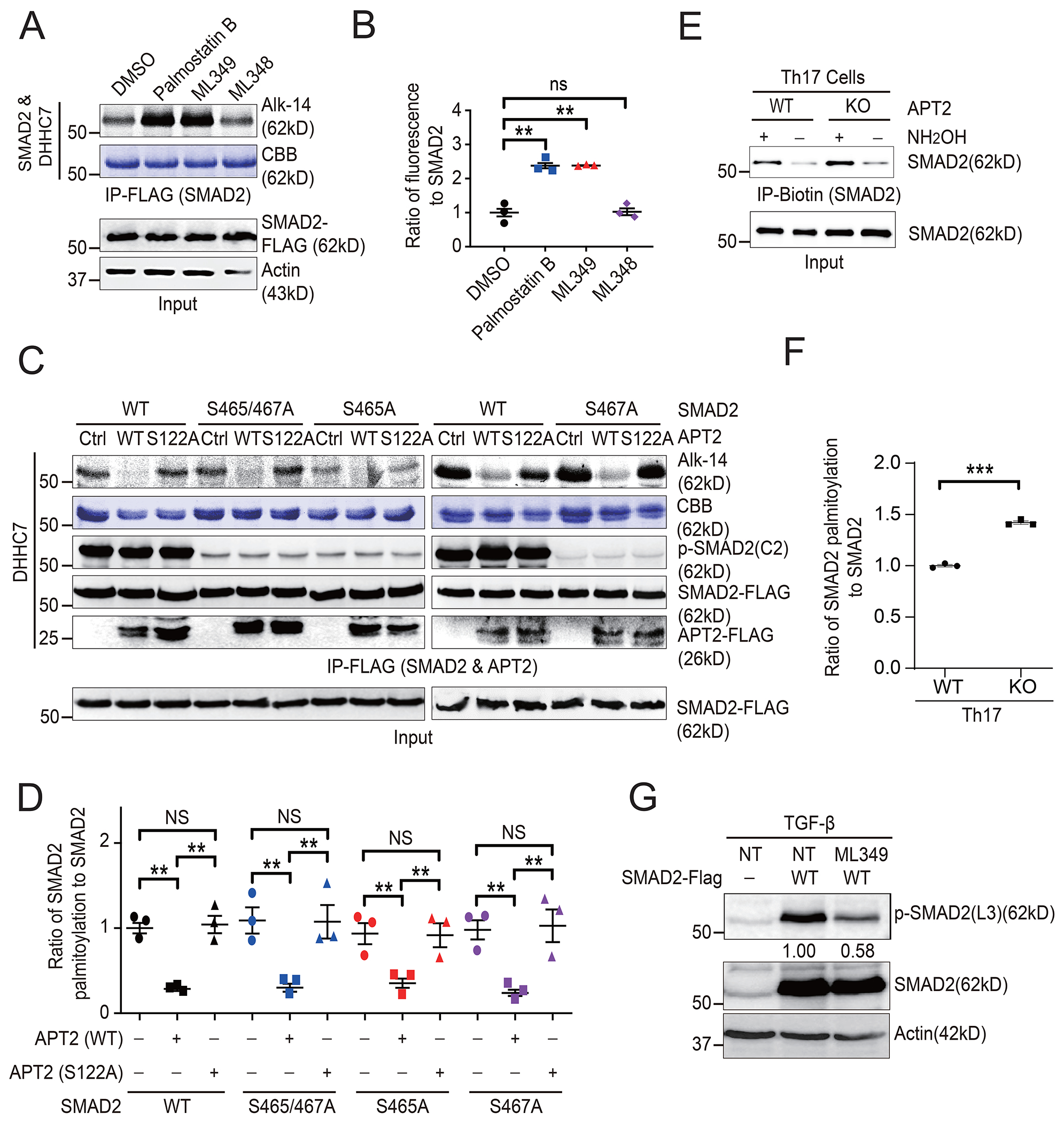
APT2 depalmitoylates SMAD2 and regulates SMAD2 linker region phosphorylation. (**A**) The palmitoylation of SMAD2 in HEK293T cells cotransfected with plasmids encoding HA-DHHC7 and FLAG-SMAD2 and treated for 24 hours with the APT inhibitors (Palmostatin B, ML349, or ML348, all at 10 μM) was detected by Alk14 labeling and in-gel fluorescence. (**B**) Relative amounts of *S*-palmitoylated SMAD2 from the experiments shown in (A) were quantified and normalized to the total amount of SMAD2 protein. (**C**) The palmitoylation of SMAD2 in DHHC7 KO HEK293T cells transfected with indicated plasmids was determined by Alk14 labeling and in-gel fluorescence, whereas the relative amounts of p-SMAD2(C2) were determined by Western blotting. (**D**) The relative amounts of *S*-palmitoylated SMAD2 from the experiments shown in (C) were quantified and normalized to the amounts of total SMAD2 protein. (**E**) The palmitoylation of SMAD2 in WT and *Lypla2*-deleted (APT2-KO) Th17 cells was determined by ABE assay. (**F**) The relative amounts of *S*-palmitoylated SMAD2 from the experiments shown in (E) were quantified and normalized to the amount s of total SMAD2 protein. (**G**) Western blotting analysis of the relative amounts of p-SMAD2(L3) in HEK293T cells transfected with plasmid encoding SMAD2-FLAG and treated with TGFβ (8 ng/ml) for 6 hours with or without treatment with 25 μM ML349 for 12 hours before detection. The relative amounts of p-SMAD2(L3) were normalized to that of total SMAD2 protein. Quantified data are means ± SEM of three experiments. **P < 0.01, ***P < 0.001; NS, not significant.

Protein *S*-palmitoylation can be reversed by acyl protein thioesterases, including APT1, APT2, and ABHD family members (23). APT2 [also known as lysophospholipase 2 (LYPLA2)] regulates STAT3 membrane localization and prefers phosphorylated STAT3 (p-STAT3) over unphosphorylated STAT3 as its substrate (8). To identify the acyl protein thioesterase mediating SMAD2 depalmitoylation, we expressed SMAD2 in HEK293T cells and treated the cells with different thioesterase inhibitors: palmostatin B (pan-depalmitoylase inhibitor), ML348 (APT1-specific inhibitor), and ML349 (APT2-specific inhibitor) (35). We found that both ML349 and palmostatin B, but not ML348, resulted in a significant increase in the abundance of *S*-palmitoylated SMAD2 (Fig. 4, A and B), suggesting that APT2 depalmitoylates palmitoylated SMAD2. To further confirm this, we co-expressed WT and the catalytically inactive S122A mutant APT2 with SMAD2 and found that WT but not mutant APT2 reduced the amount of *S*-palmitoylated SMAD2 (Fig. 4, C and D). Next, to further investigate whether APT2 mediated SMAD2 depalmitoylation in Th17 cells, we performed an ABE assay with WT and APT2 KO primary mouse Th17 cells to examine endogenous SMAD2 *S*-palmitoylation. Our data showed that APT2 depletion increased the abundance of *S*-palmitoylated SMAD2 in Th17 cells (Fig. 4, E and F), further confirming that APT2 reverses SMAD2 *S*-palmitoylation.

Subsequently, we evaluated whether APT2 prefers the pSMAD2 as a substrate. Mutation of Ser^465^, Ser^467^, or both did not affect SMAD2 depalmitoylation by APT2, implying that APT2 does not exhibit any preference between phosphorylated and unphosphorylated SMAD2 (Fig. 4, C and D). Moreover, APT2 did not significantly affect the abundance of SMAD2 phosphorylated on Ser^465^/Ser^467^ (Fig. 4, C and D). However, APT2 did regulate the phosphorylation of the linker region of SMAD2, because treatment with ML349 reduced the abundance of p-SMAD2(L3) compared to that in control cells (Fig. 4G).

### *S*-palmitoylation of SMAD2 promotes its association with SMAD4 and STAT3

**Fig. 5.**
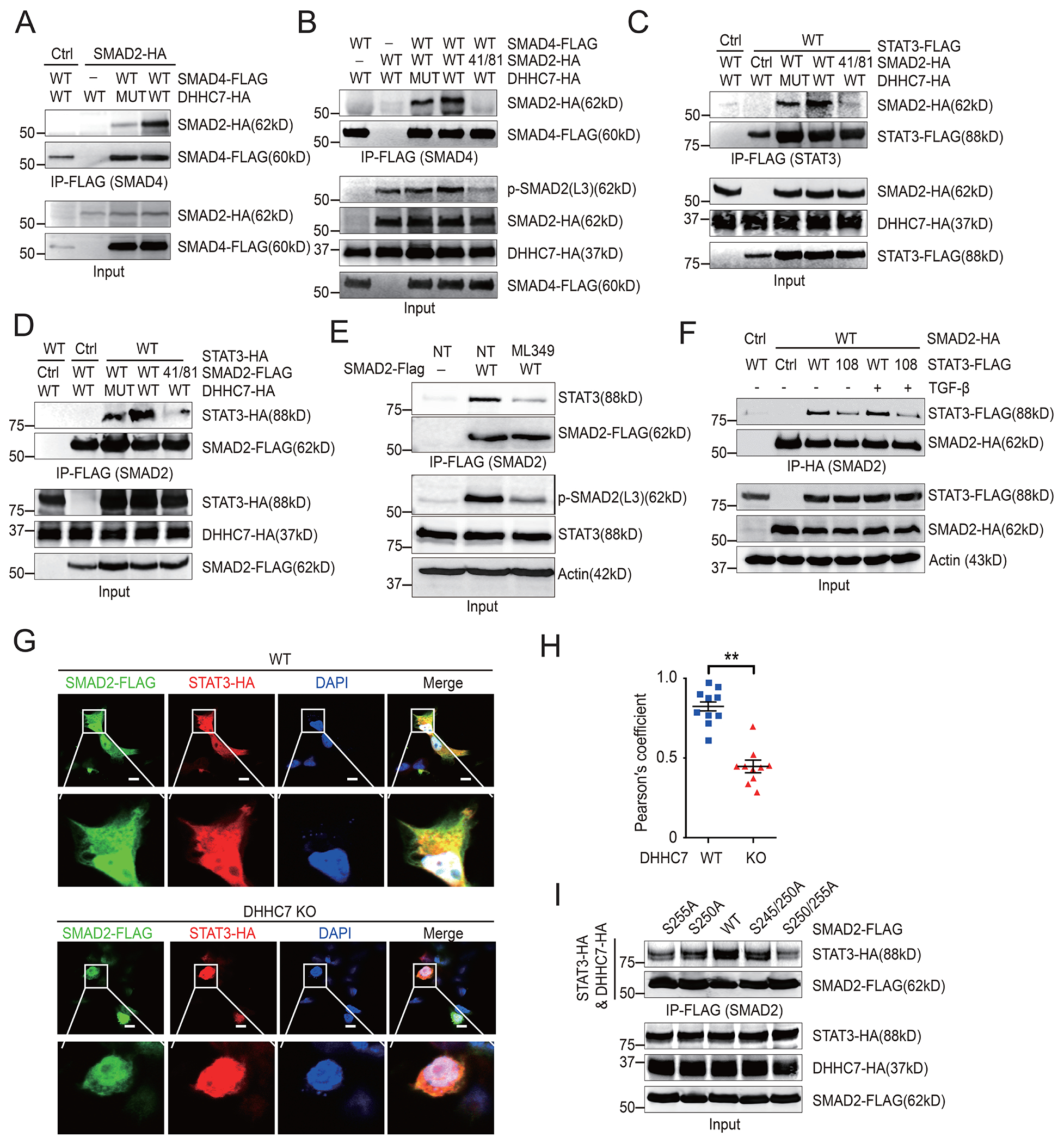
DHHC7-induced *S*-palmitoylation promotes the association of SMAD2 with SMAD4 and STAT3. (**A** and **B**) The interaction between SMAD2-HA and SMAD4-FLAG was detected by co-immunoprecipitation (Co-IP) in DHHC7 KO HEK293T cells transfected with plasmids encoding WT or catalytically inactive mutant (MUT) DHHC7-HA and the indicated SMAD2-HA and SMAD4-FLAG constructs. (**C** and **D**) The interaction between SMAD2 and STAT3 was detected by Co-IP in DHHC7 KO HEK293T cells transfected with plasmids encoding WT or catalytically inactive mutant (MUT) DHHC7 and the indicated SMAD2 and STAT3 constructs. Co-IP was performed with anti-FLAG antibody to pull down STAT3 (C) or SMAD2 (D) and assessed with anti-HA antibody. (**E**) The interaction of SMAD2 with endogenous STAT3 in HEK293T cells transfected with plasmid encoding SMAD2-FLAG and left untreated (NT) or treated with 25 μM ML349 for 12 hours. Co-IP was performed with anti-FLAG to pull down SMAD2 and assessed with anti-STAT3 antibody. (**F**) The interaction between SMAD2 and STAT3 was detected by co-IP in HEK293T cells transfected with plasmids encoding HA-tagged WT SMAD2 or the FLAG-tagged palmitoylation site Cys108Ser mutant (108) of STAT3. Cells were left untreated or were treated with TGFβ (3 ng/mL) for 6 hours. Co-IP was performed with anti-HA to pull down SMAD2 and assessed with anti-FLAG antibody to detect STAT3. (**G**) The cellular localization of SMAD2-FLAG and STAT3-HA was analyzed by confocal imaging of WT and DHHC7 KO HEK293T cells. Scale bars, 50 μm. (**H**) Colocalization of SMAD2 and STAT3 from the experiments represented in (G) was quantified using Pearson’s correlation coefficient. Each point represents a single cell from three independent experiments. (**I**) The interaction between SMAD2 and STAT3 was detected by Co-IP in HEK293T cells transfected with plasmids encoding WT STAT3-HA and WT or the linker-region phosphorylation mutants (S255A, S250A, S245/250A, S250/255A) of SMAD2-FLAG. Co-IP was performed with anti-FLAG to pull down SMAD2 and assessed with anti-HA to detect STAT3 and DHHC7. Quantified data for the experiments represented in (F) and (I) are shown in fig. S2, C and D, respectively. Data are means ± SEM of three experiments. **P < 0.01.

We next studied whether DHHC7 affected signaling downstream of SMAD2. Because the binding of SMAD4 to SMAD2 in the nucleus is important for the transcriptional activity of SMAD2 (36), we performed protein immunoprecipitation assays and found that the interaction between SMAD2 and SMAD4 was enhanced by DHHC7-catalyzed *S*-palmitoylation of SMAD2 (Fig. 5A), whereas the binding of SMAD2 to SMAD4 was decreased by expression of either catalytically inactive DHHC7 or the SMAD2 C41S/C81S mutant, which is consistent with effects on the abundance of p-SMAD2(L3) (Fig. 5B).

A previous study showed that SMAD2 phosphorylated at Ser^255^ served as a STAT3 co-activator to induce *RORC* and *IL-17A* expression (7). Because DHHC7-mediated *S*-palmitoylation promoted phosphorylation of the SMAD2 linker region, we therefore examined whether palmitoylation could facilitate the association between SMAD2 and STAT3. We found that DHHC7 promoted the SMAD2-STAT3 association, whereas expression of catalytically inactive DHHC7 or the SMAD2 C41S/C81S mutant decreased the association (Fig. 5, C and D), suggesting that DHHC7-mediated *S*-palmitoylation of SMAD2 at Cys^41^ and Cys^81^ facilitated the SMAD2-STAT3 interaction. On the other hand, treatment with ML349 reduced the abundance of p-SMAD2(L3) and, accordingly, also decreased the SMAD2-STAT3 association (Fig. 5E), suggesting that the APT2-mediated depalmitoylation of SMAD2 is also necessary for the SMAD2-STAT3 interaction. Furthermore, expression of the STAT3 C108S mutant, which blocks the palmitoylation and activation of STAT3 (8), also decreased the extent of the SMAD2-STAT3 interaction even in cells treated with TGF-β (Fig. 5F, fig. S2C), suggesting that STAT3 *S*-palmitoylation was also important for the SMAD2-STAT3 association. In addition, immunofluorescence experiments showed that more SMAD2 was colocalized with STAT3 in the cytoplasm and nucleus in WT cells than in DHHC7 KO cells, which further demonstrated that DHHC7 promoted the interaction between SMAD2 and STAT3 (Fig. 5, G and H). We next investigated which among the three phosphorylation sites in the linker region (Ser^245^/Ser^250^/Ser^255^) of SMAD2 was important for SMAD2-STAT3 complex formation. Consistent with a previous study (7), the S255A mutation resulted in significantly less SMAD2 interacting with STAT3 (Fig. 5I, fig. S2D), indicating that SMAD2 Ser^255^ phosphorylation is important for this interaction. Together, these data indicate that *S*-palmitoylation of SMAD2 facilitates its binding to STAT3 and SMAD4 by promoting phosphorylation of its linker region.

### The *S*-Palmitoylation–depalmitoylation cycle of SMAD2 promotes Th17 cell differentiation

**Fig. 6.**
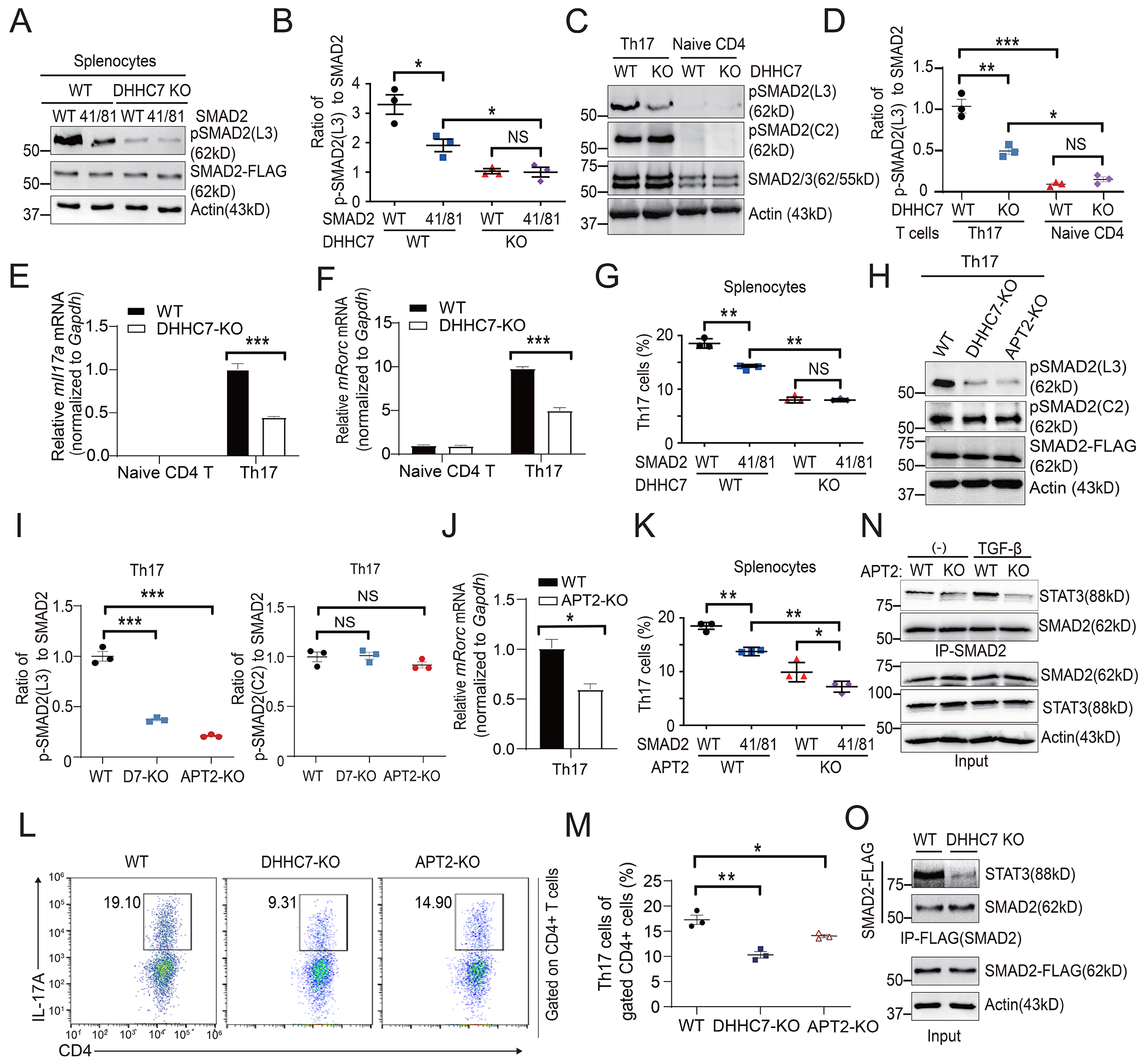
The palmitoylation-depalmitoylation cycle of SMAD2 promotes Th17 cell differentiation. (**A**) Western blotting analysis of the relative amounts of SMAD2 and p-SMAD2(L3) in WT and DHHC7 KO murine splenocytes transfected with plasmids encoding WT or Cys41/81Ser mutant (41/81) SMAD2 and cultured under T_H_17-polarizing conditions for differentiation. (**B**) The relative amounts of p-SMAD2(L3) from the experiments shown in (A) were quantified and normalized to the amount of total SMAD2 protein. (**C**) Western blotting analysis of SMAD2, p-SMAD2(L3), and p-SMAD2(C2) in naïve CD4^+^ T cells and Th17 cells from WT and DHHC7 KO mice. (**D**) The relative amounts of p-SMAD2(L3) from the experiments shown in (C) were quantified and normalized to the amount of total SMAD2 protein. (**E** and **F**) Relative amounts of *Il17a* (E) and *Rorc* (F) mRNA were detected by real-time PCR analysis of WT and DHHC7 KO naïve CD4^+^ T cells and Th17 cells. The relative amounts of *Il17a and Rorc* mRNAs were normalized to that of *Gapdh* mRNA. (**G**) Flow cytometric analysis of Th17 cell population for samples used in (A). Th17 cells were stained as CD4^+^ and IL-17^+^ cells. (**H**) The relative amounts of SMAD2, p-SMAD2(L3), and p-SMAD2(C2) in WT, DHHC7 KO, and APT2 KO mouse Th17 cells were detected by Western blotting analysis. (**I**) The relative amounts of p-SMAD2(L3) and p-SMAD2(C2) from the experiments shown in (H) were quantified and normalized to the amount of total SMAD2 protein. (**J**) The relative amounts of *Rorc* mRNA in WT and APT2 KO Th17 cells were normalized to that of *Gapdh* mRNA. (**K**) Flow cytometry analysis of Th17 cells populations within WT and APT2 KO mouse splenocytes transfected with plasmids encoding WT or Cys41/81Ser mutant (41/81) SMAD2 and cultured under Th17-polarizing conditions. (**L** and **M**) Flow cytometry analysis of the Th17 cell population for naïve CD4^+^ T cells isolated from WT, DHHC7 KO, and APT2 KO mouse spleen and cultured under Th17-polarizing conditions. Representative plots (L) and scatter graphs for quantification (M) are presented. (**N**) The interaction of endogenous STAT3 with SMAD2 in WT and APT2 KO mouse splenocytes treated without (−) or with TGFβ was detected by co-IP. Anti-SMAD2 was used for pull-downs and anti-STAT3 was used for Western blotting analysis. (**O**) The interaction of endogenous STAT3 with overexpressed SMAD2-FLAG in the splenocytes of WT and DHHC7-KO mouse was detected by co-IP. Anti-FLAG was used to pull down SMAD2, and anti-STAT3 was used for Western blotting. Quantified data are means ± SEM of three experiments. *P < 0.05, **P < 0.01; NS, not significant.

Given that SMAD2 and its association with STAT3 play a key role in Th17 cell differentiation (7, 16, 37), we studied whether SMAD2 *S*-palmitoylation regulated the differentiation of Th17 cells from naïve CD4^+^ T cells. Because previous work showed that the *S*-palmitoylation–depalmitoylation cycle of STAT3 is important for its activity and Th17 cell differentiation (8), we therefore wondered whether the same was true for SMAD2. We first determined SMAD2 phosphorylation in murine splenocytes and Th17 cells. Compared with splenocytes expressing WT SMAD2, those expressing the C41S/C81S mutant SMAD2 had decreased p-SMAD2(L3) abundance, which was further reduced in DHHC7 KO cells (Fig. 6, A and B). That loss of DHHC7 also slightly decreased the amount of the C41S/C81S mutant SMAD2 that was phosphorylated was likely because DHHC7 also regulates the *S*-palmitoylation of STAT3 (8), which may cooperate with SMAD2 palmitoylation to promote SMAD2 phosphorylation in the linker region. Similarly, loss of DHHC7 in Th17 cells also reduced the abundance of p-SMAD2(L3), but not that of p-SMAD2(C2) (Fig. 6, C and D). Consistent with the amount of p-SMAD2(L3), expression of *Il17a* and *Rorc* was significantly reduced in DHHC7 KO Th17 cells compared to that in WT Th17 cells (Fig. 6, E and F). Consequently, the differentiation of Th17 cells from the splenocytes was inhibited by either expression of the C41S/C81S mutant SMAD2 or by loss of DHHC7 (Fig. 6G and fig. S3), indicating that the DHHC7-mediated *S*-palmitoylation of SMAD2 at Cys^41^ and Cys^81^ is important for Th17 cell differentiation.

Because we hypothesized that the SMAD2 palmitoylation-depalmitoylation cycle by DHHC7 and APT2 is important for its phosphorylation and activation, we next investigated whether APT2-mediated SMAD2 depalmitoylation also regulated SMAD2 transcriptional function and Th17 cell differentiation. We first determined SMAD2 phosphorylation in APT2 KO Th17 cells and found that loss of APT2 resulted in a decrease in the abundance of p-SMAD2(L3), but not of p-SMAD2(C2), similarly to loss of DHHC7 (Fig. 6, H and I), consistent with our earlier data (Fig. 4G). Moreover, loss of APT2 inhibited the expression of *Rorc* in Th17 cells (Fig. 6J), and Th17 cell differentiation from splenocytes was also suppressed by either expression of the SMAD2 C41S/C81S mutant or knockout of APT2 (Fig. 6K). Consistent with this, the percentage of Th17 cells that differentiated from naïve CD4^+^ T cells was reduced in APT2 KO and DHHC7 KO cell cultures (Fig. 6, L and M). In addition, we also evaluated the SMAD2-STAT3 association in splenocytes and found that it was reduced in APT2 KO cells compared to that in WT cells (Fig. 6N), consistent with the suppression of Th17 cell differentiation by APT2 deletion (Fig. 6, K to M). Similarly, the SMAD2-STAT3 association was also decreased in DHHC7 KO splenocytes (Fig. 6O). Therefore, these data suggest that the palmitoylation-depalmitoylation cycle of SMAD2 regulated by DHHC7 and APT2 plays an important role in promoting SMAD2-STAT3 binding and murine primary Th17 cell differentiation.

### Inhibition of SMAD2 and STAT3 *S*-palmitoylation attenuates Th17 cell differentiation and protects mice in a multiple sclerosis model

**Fig. 7.**
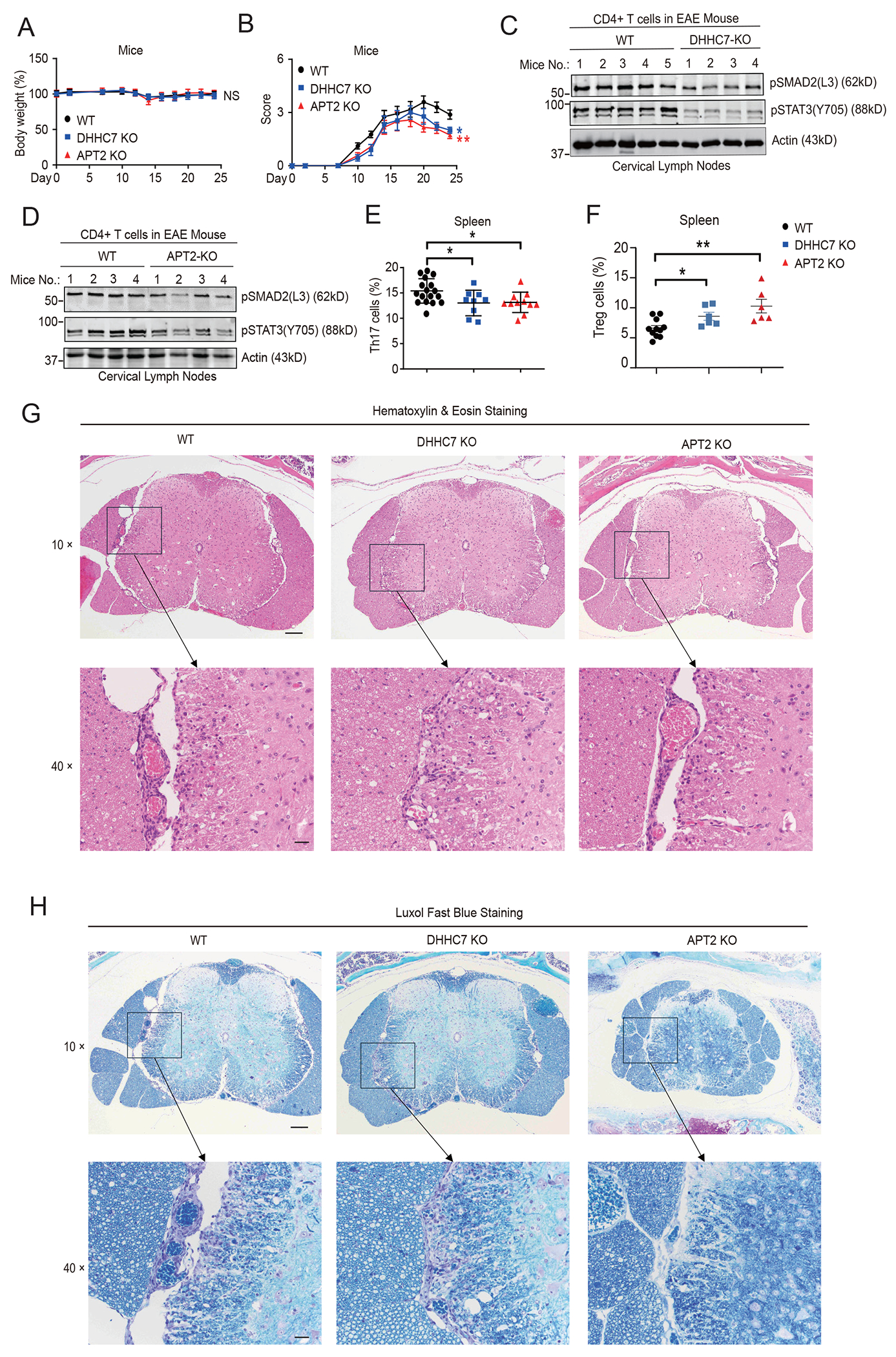
Interfering with the palmitoylation-depalmitoylation cycle of SMAD2 alleviates the severity of mice with EAE. (**A** to **H**) WT, DHHC7, and *Lypla2* KO (APT2-KO) mice were treated with MOG_35-55_ and pertussis toxin, and a second dose of pertussis toxin was used on day two after immunization. (A and B) Body weight changes (A) and clinical scores (B) were observed. (C and D) Phosphorylation of SMAD2 at its linker region [p-SMAD2(L3)] and phosphorylation of STAT3 [p-STAT3(Y705)] in CD4^+^ T cells isolated from the cervical lymph nodes of the indicated mice were analyzed by Western blotting. Quantified data for the experiments represented in (C) and (D) are shown in fig. S4, C and D, respectively. (E and F) Flow cytometry analysis of Th17 cell (E) and Treg cell (F) populations in the spleens of EAE mouse. Th17 cells were stained as CD4^+^ and IL-17^+^ cells, and Treg cells were stained as CD4^+^ and CD25^+^ Foxp3^+^ cells. (G and H) Hematoxylin & Eosin staining (G) and Luxol Fast Blue staining (H) of spinal cord sections from MOG_35-55_–immunized WT, DHHC7 KO, and APT2 KO mice to detect immune cell infiltration into the spinal cord and determine the severity of demyelination. The magnification factor is labeled at the left of the images. Scale bars, 100 μm for the 10× and 20 μm for the 40× images. Quantified data for the experiments represented in (G) and (H) are shown in fig. S4, F and G, respectively. Quantified data are means ± SEM from three individual experiments. *P < 0.05, **P < 0.01.

Th17 cells contribute to the pathogenesis of MS (38). To further test whether the palmitoylation-depalmitoylation cycles of SMAD2 and STAT3 promoted Th17 cell differentiation and aggravated inflammation in vivo, we studied the effects of loss of DHHC7 and APT2 in a myelin oligodendrocyte glycoprotein (MOG_35-55_)–induced experimental autoimmune encephalomyelitis (EAE) mouse model, which is a classical experimental model for MS (39). After administering MOG_35-55_ and pertussis toxin to the mice to induce EAE, we found no significant reduction in body weight in WT, DHHC7 KO, or APT2 KO mice (Fig. 7A, fig. S4, A and B). Subsequently, we scored disease severity and found that both DHHC7 KO mice and APT2 KO mice had a lower clinical score compared to that of WT mice (Fig. 7B). Consistent with the cellular data on SMAD2 phosphorylation (Fig. 6, C and H), both DHHC7 KO and APT2 KO EAE mice showed reduced amounts of p-SMAD2(L3) and pSTAT3 (Tyr^705^) in CD4^+^ T cells isolated from cervical lymph nodes, which are the draining lymph nodes for the central nervous system (Fig. 7, C and D, fig. S4, C and D). Moreover, compared to WT EAE mice, DHHC7 KO and APT2 KO EAE mice had decreased numbers of Th17 cells (Fig. 7E), increased numbers of Treg cells (Fig. 7F), but no change in Th1 cells (fig. S4E) in their spleens, indicating that the DHHC7- and APT2-regulated *S*-palmitoylation–depalmitoylation cycle influences the ratio of Th17 cells to Treg cells in EAE. Furthermore, loss of DHHC7 and APT2 also reduced the extent of inflammatory cell infiltration into the CNS, as well as demyelination severity in the EAE mice (Fig. 7, G and H, fig. S4 F and G). WT mice with EAE had moderate to severe white matter degeneration, myelin loss, and moderate numbers of infiltrating lymphocytes. ,Whereas similar changes were noted in DHHC7 KO mice with EAE, the degenerative changes were less severe. Additionally, the APT2 KO mice with EAE had less severe degenerative lesions in the surface white matter with reduced numbers of infiltrating lymphocytes (Fig. 7, G and H, fig. S4 F and G). Together, these findings indicate that disruption of the *S*-palmitoylation–depalmitoylation cycle of SMAD2 and STAT3 impairs the differentiation of Th17 cells and may offer a protective strategy for MS.

## DISCUSSION

**Fig. 8.**
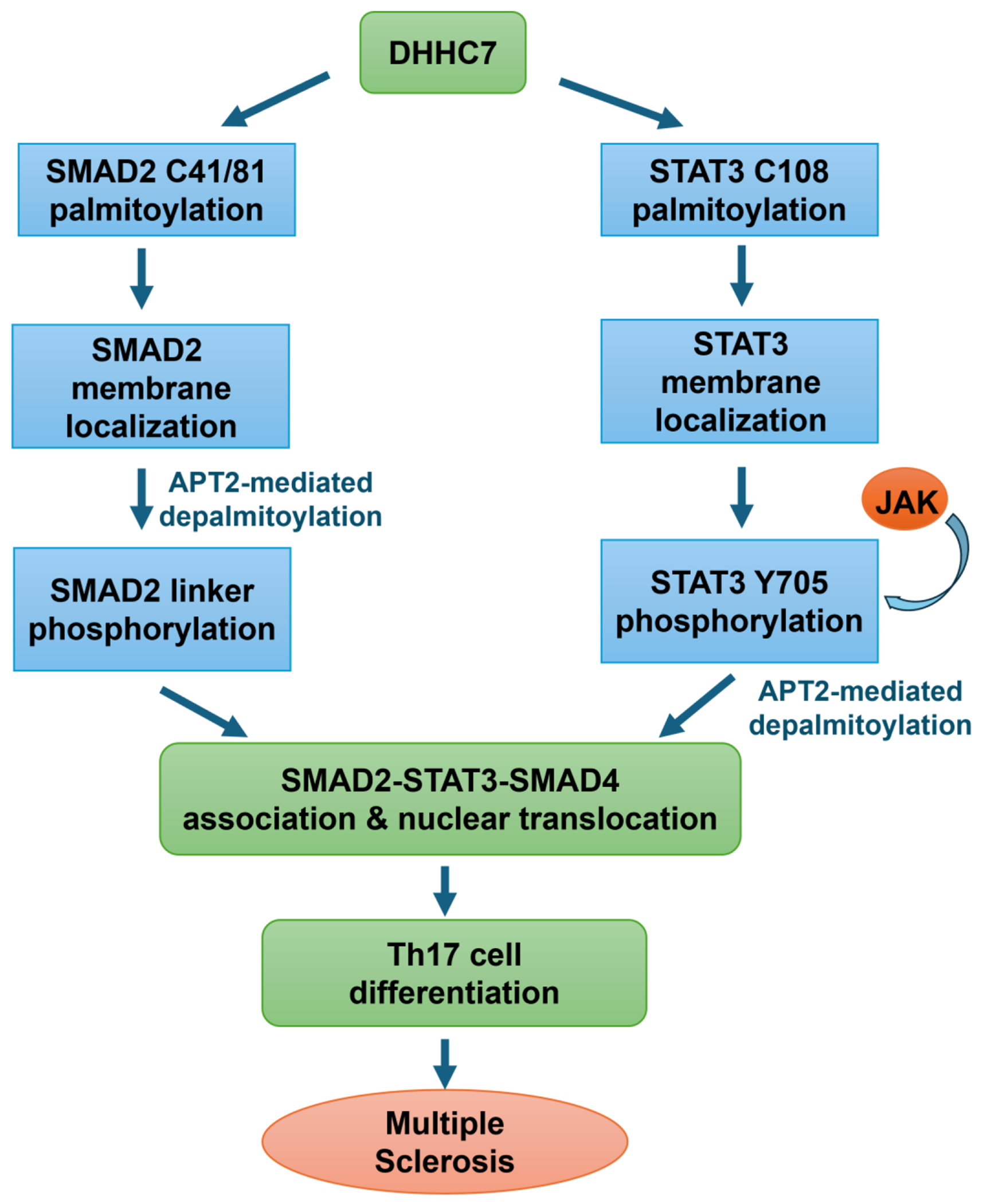
The DHHC7− and APT2-mediated palmitoylation-depalmitoylation cycle of SMAD2 and STAT3 promotes T_H_17 cell differentiation and contributes to disease severity in EAE mice. The cycle of *S*-palmitoylation of SMAD2 at Cys^41^ and Cys^81^ by DHHC7 and their depalmitoylation by APT2 facilitates the recruitment of SMAD2 to intracellular membranes to induce the phosphorylation at Ser^245^, Ser^250^, and Ser^255^ in its linker region by kinases, which promotes its association with intracellular membrane–localized STAT3 and SMAD4. DHHC7 also mediates STAT3 palmitoylation to localize STAT3 at the plasma membrane and promote its phosphorylation by JAK. APT2 then depalmitoylates p-STAT3 and promotes its association with linker-region–phosphorylated SMAD2. Subsequently, the SMAD2-STAT3-SMAD4 complex translocates to the nucleus to induce target gene expression and promote T_H_17 cell differentiation, ultimately contributing to disease severity in mice with EAE.

R-SMADs, as substrates of TGFβ receptors, undergo nuclear-cytoplasmic shuttling and play critical roles in TGFβ signaling (40). Emerging evidence suggests that posttranslational modifications have important regulatory functions in TGFβ signaling (9). For example, C-terminal tail phosphorylation is crucial for R-SMAD activation, and phosphorylation of the linker region is essential for complex formation with other co-transcription factors (7, 41). Phosphorylation of the linker region of R-SMADs was first reported to be induced by the Ras-MAPK pathway (36, 42), as well as various other kinases (11, 43–46). Although SMAD2 and SMAD3 share many common functions, they have opposing roles in T cell differentiation, with SMAD2 promoting Th17 cell differentiation, whereas SMAD3 promotes Treg cell differentiation; however, little is known about the mechanistic basis for these opposing functions (12, 15, 47, 48). Here, we found that SMAD2, but not SMAD3, was palmitoylated by DHHC7 and de-palmitoylated by APT2. This palmitoylation-depalmitoylation cycle facilitated SMAD2 phosphorylation at the linker region on the intracellular membrane (7) and its subsequent association with STAT3 (Fig. 8). This *S*-palmitoylation–depalmitoylation cycle of SMAD2 drove the differentiation of naïve T cells into Th17 cells, hence promoting inflammation and the severity of EAE.

The intracellular dynamics of *S*-palmitoylation are catalyzed by a family of 23 palmitoyl acyltransferases, known as DHHCs (22), which are integral membrane proteins localized in the endoplasmic reticulum (ER), Golgi, and plasma membrane (49). Palmitate groups can be removed by APTs (23, 24). By screening all of the DHHCs and using APT inhibitors, we revealed that DHHC7 and APT2 catalyzed SMAD2 *S*-palmitoylation and depalmitoylation, respectively, and that this SMAD2 *S*-palmitoylation–depalmitoylation cycle promoted Th17 cell differentiation. These results are consistent with a previous study showing that the activation of STAT3 is also driven by the DHHC7- and APT2-mediated *S*-palmitoylation–depalmitoylation cycle, which in turn promotes Th17 cell differentiation (8). Nevertheless, the mechanism by which SMAD2 is regulated by DHHC7 and APT2 is different from that of STAT3. DHHC7 palmitoylates STAT3 in the N terminus (Cys^108^) and promotes its membrane recruitment and phosphorylation in the C terminus (Tyr^705^), with APT2 having a higher affinity for p-STAT3 than for unphosphorylated STAT3 (8). Accordingly, APT2 depalmitoylates p-STAT3 to enable its translocation to the nucleus. However, for SMAD2, DHHC7-promoted palmitoylation does not increase the TGFβR-mediated phosphorylation of SMAD2 at the C terminus. Instead, the *S*-palmitoylation of SMAD2 promotes its phosphorylation at its linker region which occurs mainly on intracellular membranes (7). Moreover, the APT2-mediated depalmitoylation of SMAD2 is also necessary for linker region phosphorylation and its consequent association with STAT3. Membrane trafficking regulates SMAD signaling (50, 51). A possible explanation for the observation that the DHHC7- and APT2-catalyzed palmitoylation cycle promotes SMAD2 linker region phosphorylation is that the cycle promotes its endocytic trafficking, which is required for the linker region phosphorylation. Additionally, unlike for STAT3, APT2 has no preference for p-SMAD2 (Ser^465^/Ser^467^) as compared to unphosphorylated SMAD2.

Given that SMAD2 phosphorylated at its linker region can form a complex with STAT3 to promote Th17 cell differentiation (7), our finding further showcases that this process is promoted by the *S*-palmitoylation–depalmitoylation cycle. By enhancing the SMAD2-STAT3 association, DHHC7 and APT2 promote Th17 cell differentiation. We also confirmed that Ser^255^ is the major phosphorylation site of SMAD2 and that it promotes the binding of SMAD2 to STAT3. Our study supports the idea that an *S*-palmitoylation–depalmitoylation cycle, which is controlled by DHHC7 and APT2, promotes SMAD2-STAT3-SMAD4 complex formation and Th17 cell differentiation.

Although SMAD2 and SMAD3 are highly homologous and share the same upstream signaling pathway, as well as many downstream signaling events, they opposingly promote the differentiation of Th17 cells and Treg cells, respectively (7, 12, 15). Even though previous reports elucidated the important role of SMAD2 linker region phosphorylation in promoting Th17 cell differentiation (7, 45, 46), how T cells control this phosphorylation event is an unaddressed question. Based on sequence alignments, the linker phosphorylation sites are conserved between SMAD2 and SMAD3, but one of the palmitoylation sites of SMAD2, Cys^81^, is not present in SMAD3. Therefore, our research indicates that *S*-palmitoylation at Cys^81^ (and Cys^41^), which is present in an unstructured loop of SMAD2 but not present in SMAD3 (figs. S5 and S6), is a major determining factor for the phosphorylation of SMAD2 at the linker region and for the opposing roles of SMAD2 and SMAD3 in T cell differentiation. Accordingly, it is reasonable to postulate that in T cells with more active DHHC7, SMAD2 will be palmitoylated and phosphorylated at the linker region, bind to STAT3, and promote Th17 cell differentiation. In contrast, when there is no active DHHC7, the interaction between SMAD2 and STAT3 will not be promoted, and Th17 cell differentiation will be repressed.

TGFβ-activated SMAD2 acts as an important transcriptional factor in proinflammatory Th17 cells (7, 38, 46), which are abundant in patients with MS and further increase in number during relapses (38, 52). Thus, cells from the Th17 axis represent a major target of MS therapeutics (38). Interestingly, no differential expression of *SMAD2*, *SMAD3*, or *SMAD4* was observed in circulating CD4^+^ T cells from patients with MS compared to those from healthy controls (53). This implies that posttranslational regulation of R-SMADs plays an important role in MS pathogenesis. Indeed, our study found that *S*-palmitoylation promoted SMAD2 activation and Th17 cell differentiation, contributing to pathogenesis in mice with EAE, a model of MS. Previous research also showed that STAT3 activation is driven by the DHHC7- and APT2-catalyzed *S*-palmitoylation–depalmitoylation cycle (8). Together, these results suggest that targeting the *S*-palmitoylation–depalmitoylation cycles of SMAD2 and STAT3 could be a potential therapeutic strategy for MS treatment. Furthermore, given that Th17 cells play an important role in various human autoimmune diseases, including inflammatory bowel disease (8, 54), arthritis (55), type I diabetes (56), and graft-versus-host disease (57), this DHHC7- and APT2-controlled *S*-palmitoylation–depalmitoylation cycle may provide new therapeutic strategies for various human autoimmune diseases.

## MATERIALS AND METHODS

### Antibodies and common reagents

Antibodies specific for SMAD3 (ab28379), p-SMAD3 (S423/S425) (ab52903), SMAD2 (ab40855), Na/K-ATPase (ab254025), DHHC7 (ab138210), and FLAG (ab1162, ab18230) were purchased from Abcam. Antibodies against SMAD3 (sc-101154) and the HA-probe (SC7392 and SC805) were obtained from Santa Cruz. Antibody against histone H3 (4499S), Alexa Fluor 350–conjugated goat anti-rabbit IgG (A-11046), Alexa Fluor 594–conjugated goat anti-mouse IgG (8890S), anti-mouse IgG HRP (7076S), and anti-rabbit IgG HRP (7074S) were bought from CST. Mouse CD4 PerCP-Cy5.5 and Mouse Foxp3 Alexa Fluor 647 were purchased from BD Pharmingen. Antibodies against DHHC7 (R12-3691) and β-Actin (AP0060) and Alexa Fluor 350–conjugated goat anti-rabbit IgG (A-11046) were also used. Other reagents were purchased from commercial sources, including ML348 (S6564, Selleckchem), ML349 (HY-100737, MedChemExpress), palmostatin B (178501, Sigma-Aldrich), Protease inhibitor cocktail (68298, Roche), Fedratinib (S2736, Selleckchem), Universal nuclease (88700, Thermo Fisher Scientific), Bradford assay (23200, Thermo Fisher Scientific), dithiothreitol (DTT) (DTT100, Gold Biotechnology), Enzyme-linked chemiluminescence (ECL) plus (32132, Thermo Fisher Scientific), SYBR Green PCR Master Mix (4472908, Thermo Fisher Scientific). Streptavidin Agarose (20359, Thermo Fisher Scientific), Protein A/G PLUS-Agarose (sc-2003, Santa Cruz), Anti-FLAG Agarose Gel (A2220, Sigma-Aldrich), and Anti-HA Affinity Gel (E6779, Sigma-Aldrich).

### *DHHC7* knockout mice

The mouse strain B6.129P2(FVB)-DHHC7tm1.2Lusc/Mmmh, RRID: MMRRC_043511-MU was obtained from the Mutant Mouse Resource and Research Center (MMRRC) at the University of Missouri, an NIH-funded strain repository, and was donated to the MMRRC by Bernhard Luscher, Ph.D., The Pennsylvania State University. For genotype identification, we used primers for the WT (forward: TGAGCCAGGATGGATTTCAGACA; backward: TGCCCTCGGACGCAGGAGATGAA) and primers for the mutant (forward: TCCCCTGATGTATGCGAATGTCC; backward: AACAGGTGCCTTTTGAATGTCAG).

### *Lypla2* knockout mice

The *Lypla2* knockout mouse was generated by the Cornell Stem Cell & Transgenic Core Facility using the same mouse strain as the *DHHC7* knockout mouse at Cornell University. *Lypla2* knockout mice were generated as previously described (58). The design of the guide RNA (gRNA) was performed with the CRISPR Design Tool (bioinfo and ExonPrimer) to minimize potential off-target effects. The following gRNA sequences were used: 5′- CTGGAACGGAAGTTCCGGCGGGG -3′ and 5′- AGCGGGAAACGGCCGCGGTAAGG -3′. For genotype identification, we used primers for the WT (forward: CTTCTCTCCCCTGGCTTTG; backward: TAGGACCAGAGGACAACCCC) and primers for the mutant (forward: GGCAACATGGCAGCTTCTAC; backward: TAGGACCAGAGGACAACCCC.)

### Mouse model of multiple sclerosis (MS)

The mouse protocol 2019-0009 was approved by Cornell University’s Institutional Animal Care and Use Committee (IACUC). All animals were housed under specific pathogen free (SPF) conditions according to the regulations of Cornell University’s IACUC. Mice (6- to 8-weeks old) mice were randomized into different groups (at least nine mice per group with mixed sexes). Myelin oligodendrocyte glycoprotein (MOG35-55)–induced EAE was generated as previously described (39, 59–61). MS scores and body weight were monitored every other day. All mice were euthanized, and the spleens and cervical lymph nodes were isolated to detect T cells. Spinal cords were transcardially perfused with 4% paraformaldehyde (PFA), then fixed in 4% PFAovernight, dehydrated with ethanol, and embedded in paraffin, followed by hematoxylin & eosin (H&E) staining and Luxol Fast Blue Staining, which were performed as previously described (59).

### Cloning and mutagenesis

Plasmids encoding murine DHHC 1-23 were obtained from Professor Masaki Fukata, National Institutes of Natural Sciences of Japan. Plasmid encoding APT2 was obtained from GenScript. Expression vectors encoding STAT3 and SMADs with different tags were purchased from Addgene (catalog 8706 for mouse STAT3, catalog 8709 for mouse STAT3-Y705F, catalog 14930 for human SMAD2, catalog 11742 for human SMAD3, catalog 16483 for human SMAD4, and catalog 80888 for human SMAD4). Site-directed mutagenesis using these plasmids was performed by Quik Change site-directed mutagenesis, as described previously (62).

### Cell isolation, differentiation, and transfection

Human HEK293T cells were cultured in DMEM (11965–092, Thermo Fisher Scientific), supplied with 10% fetal bovine serum (FBS, 26140079, FBS, 26140079). *DHHC7* KO HEK293T cells were generated as previously described (8). The knockout of *DHHC7* was subsequently verified by Western blotting analysis. Naive CD4^+^ T cells were extracted from mouse spleens as described previously (54) and were cultured in anti-CD3/CD28–coated plates with 3 ng/ml TGF-β (100-21, Thermo Fisher Scientific), 40 ng/ml IL-6 (200-06, Thermo Fisher Scientific), 30 ng/ml IL-23 (200-23, Thermo Fisher Scientific), 20 ng/ml Tumor Necrosis Factor (TNF)-α (300-01A, Thermo Fisher Scientific), and 10 ng/ml IL-1β (200-01B, Thermo Fisher Scientific). Transient transfections were performed with either FuGene 6 (E2691, Promega) (for HEK293T cells) or the Gene Pulser Xcell^™^ system (1652677, Bio-Rad) (for splenocytes).

### *S*-palmitoylation detection

Click chemistry and in-gel fluorescence detection and Acyl-biotin exchange (ABE) were used for *S*-palmitoylation detection as described previously (8, 19).

### Subcellular fractionation and Western blotting analysis

Subcellular fractionations and Western blotting analysis were performed as previously described (8, 63, 63–65). After harvesting, cells were suspended in subcellular fraction buffer [250 mM sucrose, 20 mM HEPES (pH 7.4), 10 mM KCl, 1.5 mM MgCl_2_, 1 mM EDTA, 1 mM EGTA and 1 mM DTT) with protease inhibitor cocktail and nuclease and homogenized with a 25-gauge syringe needle. The cells were then lysed for 30 min on ice. The cell lysate was separated by centrifugation at 1000g for 5 min, and the pellet was used as the nuclear fraction. The supernatant was further separated by centrifugation at 6000g for 5 min to remove the mitochondria, and the resulting supernatant was then centrifugated at 20,000g for 2 hours to obtain the cytosolic (supernatant, which includes intracellular membranes) and plasma membrane (pellet) fractions. All of the fractions were dissolved in 4% SDS lysis buffer [4% SDS, 50 mM triethanolamine (pH 7.4), and 150 mM NaCl). After BCA assays were performed, normalized portions of different fractions were subjected to Western blotting analysis. Proteins were detected by chemiluminescence with ECL plus on a Typhoon scanner, according to the manufacturer’s instructions.

### Immunofluorescence microscopy

Immunofluorescence microscopy was conducted as previously described (8, 19, 63). Cells were seeded in 35-mm glass bottom dishes (MatTek) and fixed with 4% PFA (v/v in PBS) for 30 min. The fixed cells were washed twice with PBS and then permeabilized and blocked with 0.1% Saponin/5% BSA/PBS for 30 min. The permeabilized cells were incubated overnight at 4°C in the dark with primary antibodies, which was followed by incubation with the appropriate secondary antibodies (Alexa Fluor 350–conjugated goat anti-rabbit IgG and Alexa Fluor 594–conjugated goat anti-mouse IgG) at room temperature in the dark for 1 hour. Samples were mounted with Fluoromount-G (0100-01) or DAPI Fluoromount-G (0100-20) and observed with an inverted confocal microscopy (LSM880). Quantification of fluorescence images was performed with Image J software (for Figs. 2E, 3H, and 5H) or Cell Profiler (for Fig. 2H).

### Quantitative real-time PCR (qPCR)

To analyze gene expression, qPCR was performed with the SYBR Green PCR Master Mix according to standard procedures. The sequences of gene-specific primers used for qPCR analysis were published previously (7, 8, 19).

### Flow cytometry analysis and sorting

Flow cytometry was performed according to previous procedures with moderate optimization (8, 61, 63). Briefly, for staining of Treg cells, 1 × 10^6^ cells per sample were fixed and labeled with antibodies (anti-CD16/32a for Fc Receptor blocking, Violet Dye for living cells, and anti-CD4, anti-CD25 and anti-Foxp3 for the Treg cell population). For Th17 cell staining, 1 × 10^6^ cells per sample were fixed and labeled with antibodies (anti-CD16/32a, Violet Dye, and anti-CD4). After washing, permeabilization, and fixation, the cells were labeled with anti-IL-17A antibody to detect the Th17 cell population. Each sample was then processed with an Attune Flow Cytometer and analyzed with Flowjo v10.8 software. The percentage of T_reg_ cells were determined as CD25^+^FoxP3^+^ after CD45^+^ CD4^+^ T cells were gated, and the percentage of T_H_17 cells were determined as IL-17^+^ CD4^+^ T cells after gating on CD45^+^ cells.

### Statistical analysis

SPSS 17.0 was used for data analysis. All data are presented as means ± the standard error of the mean (SEM). Differences between groups were tested with the student’s t test. Other data was analyzed by one-way analysis of variance (ANOVA).

## Supplementary Material

main supplementary

MDAR checklist

## Data Availability

All data needed to evaluate the conclusions in the paper are present in the paper or the Supplementary Materials.

## References

[1] Ouyang W, Beckett O, Ma Q, Li MO, Transforming Growth Factor-β Signaling Curbs Thymic Negative Selection Promoting Regulatory T Cell Development. Immunity 32, 642–653 (2010). 20471291 10.1016/j.immuni.2010.04.012 PMC2880228

[2] Tato CM, O’Shea JJ, Immunology: what does it mean to be just 17? Nature 441, 166–168 (2006). 16688162 10.1038/441166a

[3] Gutcher I, Donkor MK, Ma Q, Rudensky AY, Flavell RA, Li MO, Autocrine transforming growth factor-beta1 promotes in vivo Th17 cell differentiation. Immunity 34, 396–408 (2011). 21435587 10.1016/j.immuni.2011.03.005 PMC3690311

[4] David CJ, Massagué J, Contextual determinants of TGFβ action in development, immunity and cancer. Nature Reviews Molecular Cell Biology 19, 419–435 (2018). 29643418 10.1038/s41580-018-0007-0 PMC7457231

[5] Ivanov BSM II, Zhou L, Tadokoro CE, Lepelley A, Lafaille JJ, Cua DJ, Littman DR, The orphan nuclear receptor RORgammat directs the differentiation program of proinflammatory IL-17+ T helper cells. Cell 126, 1121–1133 (2006). 16990136 10.1016/j.cell.2006.07.035

[6] Duscha A, Gisevius B, Hirschberg S, Yissachar N, Stangl GI, Eilers E, Bader V, Haase S, Kaisler J, David C, Schneider R, Troisi R, Zent D, Hegelmaier T, Dokalis N, Gerstein S, Mare-Roumani SD, Amidror S, Staszewski O, Poschmann G, Stuhler K, Hirche F, Balogh A, Kempa S, Trager P, Zaiss MM, Holm JB, Massa MG, Nielsen HB, Faissner A, Lukas C, Gatermann SG, Scholz M, Przuntek H, Prinz M, Forslund SK, Winklhofer KF, Muller DN, Linker RA, Gold R, Haghikia A, Propionic Acid Shapes the Multiple Sclerosis Disease Course by an Immunomodulatory Mechanism. Cell 180, 1067–1080 1016 (2020). 32160527 10.1016/j.cell.2020.02.035

[7] Yoon JH, Sudo K, Kuroda M, Kato M, Lee IK, Han JS, Nakae S, Imamura T, Kim J, Ju JH, Kim DK, Matsuzaki K, Weinstein M, Matsumoto I, Sumida T, Mamura M, Phosphorylation status determines the opposing functions of Smad2/Smad3 as STAT3 cofactors in TH17 differentiation. Nat Commun 6, 7600 (2015). 26194464 10.1038/ncomms8600 PMC4518312

[8] Zhang M, Zhou L, Xu Y, Yang M, Xu Y, Komaniecki GP, Kosciuk T, Chen X, Lu X, Zou X, Linder ME, Lin H, A STAT3 palmitoylation cycle promotes TH17 differentiation and colitis. Nature 586, 434–439 (2020). 33029007 10.1038/s41586-020-2799-2 PMC7874492

[9] Matsuzaki K, Smad phospho-isoforms direct context-dependent TGF-β signaling. Cytokine & Growth Factor Reviews 24, 385–399 (2013). 23871609 10.1016/j.cytogfr.2013.06.002

[10] Chaikuad A, Bullock AN, Structural Basis of Intracellular TGF-β Signaling: Receptors and Smads (2016). 10.1101/cshperspect.a022061. PMC5088531 27549117

[11] Souchelnytskyi S, Tamaki K, Engstrom U, Wernstedt C, Dijke P, Heldin CH, Phosphorylation of Ser465 and Ser467 in the C terminus of Smad2 mediates interaction with Smad4 and is required for transforming growth factor-beta signaling. J Biol Chem 272, 28107–28115 (1997). 9346966 10.1074/jbc.272.44.28107

[12] Joetham A, Schedel M, Ning F, Wang M, Takeda K, Gelfand EW, Dichotomous role of TGF-beta controls inducible regulatory T-cell fate in allergic airway disease through Smad3 and TGF-beta-activated kinase 1. J Allergy Clin Immunol 145, 933–946 934 (2020). 31626843 10.1016/j.jaci.2019.09.032 PMC11098441

[13] Liu X, Sun Y, Constantinescu SN, Karam E, Weinberg RA, Lodish HF, Transforming growth factor beta-induced phosphorylation of Smad3 is required for growth inhibition and transcriptional induction in epithelial cells. Proc Natl Acad Sci U S A 94, 10669–10674 (1997). 9380693 10.1073/pnas.94.20.10669 PMC23442

[14] Shi Y, Hata A, Lo RS, Massague J, Pavletich NP, A structural basis for mutational inactivation of the tumour suppressor Smad4. Nature 388, 87–93 (1997). 9214508 10.1038/40431

[15] Kashiwagi I, Morita R, Schichita T, Komai K, Saeki K, Matsumoto M, Takeda K, Nomura M, Hayashi A, Kanai T, Yoshimura A, Smad2 and Smad3 Inversely Regulate TGF-beta Autoinduction in Clostridium butyricum-Activated Dendritic Cells. Immunity 43, 65–79 (2015). 26141582 10.1016/j.immuni.2015.06.010

[16] Zhang S, The role of transforming growth factor β in T helper 17 differentiation. Immunology 155, 24–35 (2018). 29682722 10.1111/imm.12938 PMC6099164

[17] Linder ME, Deschenes RJ, Palmitoylation: policing protein stability and traffic. Nature Reviews Molecular Cell Biology 8, 74–84 (2007). 17183362 10.1038/nrm2084

[18] Lin H, Protein cysteine palmitoylation in immunity and inflammation. The FEBS Journal 288, 7043–7059 (2021). 33506611 10.1111/febs.15728 PMC8872633

[19] Yu T, Hou D, Zhao J, Lu X, Greentree WK, Zhao Q, Yang M, Conde D-G, Linder ME, Lin H, NLRP3 Cys126 palmitoylation by ZDHHC7 promotes inflammasome activation. Cell Reports 43 (2024). 10.1016/j.celrep.2024.114070 PMC11130711 38583156

[20] Zhang B, Mullmann J, Ludewig AH, Fernandez IR, Bales TR, Weiss RS, Schroeder FC, Acylspermidines are conserved mitochondrial sirtuin-dependent metabolites. Nature Chemical Biology, doi: 10.1038/s41589-023-01511-2 (2024). PMC11715332 38167917

[21] Zhang B, Yu Y, Fox BW, Liu Y, Thirumalaikumar VP, Skirycz A, Lin H, Schroeder FC, Amino acid and protein specificity of protein fatty acylation in *Caenorhabditis elegans*. Proceedings of the National Academy of Sciences 121, e2307515121 (2024). 10.1073/pnas.2307515121 PMC10835129 38252833

[22] Linder ME, Jennings BC, Mechanism and function of DHHC S-acyltransferases. Biochem Soc Trans 41, 29–34 (2013). 23356254 10.1042/BST20120328

[23] Dekker FJ, Rocks O, Vartak N, Menninger S, Hedberg C, Balamurugan R, Wetzel S, Renner S, Gerauer M, Scholermann B, Rusch M, Kramer JW, Rauh D, Coates GW, Brunsveld L, Bastiaens PI, Waldmann H, Small-molecule inhibition of APT1 affects Ras localization and signaling. Nat Chem Biol 6, 449–456 (2010). 20418879 10.1038/nchembio.362

[24] Duncan JA, Gilman AG, A cytoplasmic acyl-protein thioesterase that removes palmitate from G protein alpha subunits and p21(RAS. J Biol Chem 273, 15830–15837 (1998). 9624183 10.1074/jbc.273.25.15830

[25] Greaves J, Chamberlain LH, DHHC palmitoyl transferases: substrate interactions and (patho)physiology. Trends Biochem Sci 36, 245–253 (2011). 21388813 10.1016/j.tibs.2011.01.003

[26] Jiang H, Zhang X, Chen X, Aramsangtienchai P, Tong Z, Lin H, Protein Lipidation: Occurrence, Mechanisms, Biological Functions, and Enabling Technologies. Chem. Rev 118, 919–988 (2018). 29292991 10.1021/acs.chemrev.6b00750 PMC5985209

[27] Zhang Y, Qin Z, Sun W, Chu F, Zhou F, Function of Protein S-Palmitoylation in Immunity and Immune-Related Diseases. Frontiers in Immunology 12 (2021). 10.3389/fimmu.2021.661202 PMC8453015 34557182

[28] Li N, Yang Y, He K, Zhang F, Zhao L, Zhou W, Yuan J, Liang W, Fang X, Single-Molecule Imaging Reveals the Activation Dynamics of Intracellular Protein Smad3 on Cell Membrane. Sci Rep 6, 33469 (2016). 27641076 10.1038/srep33469 PMC5027577

[29] Hough C, Radu M, Doré JJE, TGF-Beta Induced Erk Phosphorylation of Smad Linker Region Regulates Smad Signaling. PLOS ONE 7, 1–10 (2012). 10.1371/journal.pone.0042513 PMC3412844 22880011

[30] Ohnishi H, Miyata T, Yasuda H, Satoh Y, Hanatsuka K, Kita H, Ohashi A, Tamada K, Makita N, Iiri T, Ueda N, Mashima H, Sugano K, Distinct Roles of Smad2-, Smad3-, and ERK-dependent Pathways in Transforming Growth Factor-β1 Regulation of Pancreatic Stellate Cellular Functions*. Journal of Biological Chemistry 279, 8873–8878 (2004). 14688282 10.1074/jbc.M309698200

[31] Aramsangtienchai P, Spiegelman NA, Cao J, Lin H, S-Palmitoylation of Junctional Adhesion Molecule C Regulates Its Tight Junction Localization and Cell Migration. J Biol Chem 292, 5325–5334 (2017). 28196865 10.1074/jbc.M116.730523 PMC5392678

[32] Jiang Y, Xu Y, Zhu C, Xu G, Xu L, Rao Z, Zhou L, Jiang P, Malik S, Fang J, Lin H, Zhang M, STAT3 palmitoylation initiates a positive feedback loop that promotes the malignancy of hepatocellular carcinoma cells in mice. Science Signaling 16, eadd2282 (2023). 38051779 10.1126/scisignal.add2282 PMC10907978

[33] Jiang S, Zhao X, Chen S, Pan G, Song J, He N, Li F, Cui W, Fan C, Down-regulating ERK1/2 and SMAD2/3 phosphorylation by physical barrier of celecoxib-loaded electrospun fibrous membranes prevents tendon adhesions. Biomaterials 35, 9920–9929 (2014). 25201739 10.1016/j.biomaterials.2014.08.028

[34] Wrighton KH, Lin X, Feng XH, Phospho-control of TGF-beta superfamily signaling. Cell Res 19, 8–20 (2009). 19114991 10.1038/cr.2008.327 PMC2929013

[35] Kathayat RS, Elvira PD, Dickinson BC, A fluorescent probe for cysteine depalmitoylation reveals dynamic APT signaling. Nat Chem Biol 13, 150–152 (2017). 27992880 10.1038/nchembio.2262 PMC5247352

[36] Massague J, Integration of Smad and MAPK pathways: a link and a linker revisited. Genes Dev 17, 2993–2997 (2003). 14701870 10.1101/gad.1167003

[37] Malhotra N, Robertson E, Kang J, SMAD2 Is Essential for TGFβ-mediated Th17 Cell Generation *[S]. Journal of Biological Chemistry 285, 29044–29048 (2010). 20656683 10.1074/jbc.C110.156745 PMC2937934

[38] Moser T, Akgun K, Proschmann U, Sellner J, Ziemssen T, The role of TH17 cells in multiple sclerosis: Therapeutic implications. Autoimmun Rev 19, 102647 (2020). 32801039 10.1016/j.autrev.2020.102647

[39] Bittner S, Afzali AM, Wiendl H, Meuth SG, Myelin Oligodendrocyte Glycoprotein (MOG_35-55_) Induced Experimental Autoimmune Encephalomyelitis (EAE) in C57BL/6 Mice. JoVE, 51275 (2014). 24797125 10.3791/51275 PMC4172026

[40] Hata A, Chen Y-G, TGF-β Signaling from Receptors to Smads (2016). 10.1101/cshperspect.a022061 PMC5008074 27449815

[41] Fransvea E, Mazzocca A, Antonaci S, Giannelli G, Targeting transforming growth factor (TGF)-βRI inhibits activation of β1 integrin and blocks vascular invasion in hepatocellular carcinoma. Hepatology 49, 839–850 (2009). 19115199 10.1002/hep.22731

[42] Kretzschmar M, Doody J, Massagu J, Opposing BMP and EGF signalling pathways converge on the TGF-β family mediator Smad1. Nature 389, 618–622 (1997). 9335504 10.1038/39348

[43] Funaba M, Zimmerman CM, Mathews LS, Modulation of Smad2-mediated Signaling by Extracellular Signal-regulated Kinase *. Journal of Biological Chemistry 277, 41361–41368 (2002). 12193595 10.1074/jbc.M204597200

[44] van Caam A, Madej W, Garcia de Vinuesa A, Goumans M-J, ten Dijke P, Blaney Davidson E, van der Kraan P, TGFβ1-induced SMAD2/3 and SMAD1/5 phosphorylation are both ALK5-kinase-dependent in primary chondrocytes and mediated by TAK1 kinase activity. Arthritis Research & Therapy 19, 112 (2017). 28569204 10.1186/s13075-017-1302-4 PMC5452635

[45] Liang J, Zhou Y, Zhang N, Wang D, Cheng X, Li K, Huang R, Lu Y, Wang H, Han D, Wu W, Han M, Miao S, Wang L, Zhao H, Song W, The phosphorylation of the Smad2/3 linker region by nemo-like kinase regulates TGF-β signaling. J Biol Chem 296, 100512 (2021). 33676893 10.1016/j.jbc.2021.100512 PMC8047224

[46] Kamato D, Little PJ, Smad2 linker region phosphorylation is an autonomous cell signalling pathway: Implications for multiple disease pathologies. Biomedicine & Pharmacotherapy 124, 109854 (2020). 31981946 10.1016/j.biopha.2020.109854

[47] Piek E, Ju WJ, Heyer J, Escalante-Alcalde D, Stewart CL, Weinstein M, Deng C, Kucherlapati R, Böttinger EP, Roberts AB, Functional Characterization of Transforming Growth Factor β Signaling in Smad2- and Smad3-deficient Fibroblasts*. Journal of Biological Chemistry 276, 19945–19953 (2001). 11262418 10.1074/jbc.M102382200

[48] Brown KA, Pietenpol JA, Moses HL, A tale of two proteins: Differential roles and regulation of Smad2 and Smad3 in TGF-β signaling. Journal of Cellular Biochemistry 101, 9–33 (2007). 17340614 10.1002/jcb.21255

[49] Gorleku OA, Barns AM, Prescott GR, Greaves J, Chamberlain LH, Endoplasmic reticulum localization of DHHC palmitoyltransferases mediated by lysine-based sorting signals. J Biol Chem 286, 39573–39584 (2011). 21926431 10.1074/jbc.M111.272369 PMC3234780

[50] Chung C-L, Tai S-B, Hu T-H, Chen J-J, Chen C-L, Roles of Myosin-Mediated Membrane Trafficking in TGF-β Signaling. International Journal of Molecular Sciences 20 (2019). 10.3390/ijms20163913 PMC6719161 31408934

[51] Panopoulou E, Gillooly DJ, Wrana JL, Zerial M, Stenmark H, Murphy C, Fotsis T, Early Endosomal Regulation of Smad-dependent Signaling in Endothelial Cells*. Journal of Biological Chemistry 277, 18046–18052 (2002). 11877415 10.1074/jbc.M107983200

[52] Dendrou CA, Fugger L, Friese MA, Immunopathology of multiple sclerosis. Nature Reviews Immunology 15, 545–558 (2015). 10.1038/nri3871 26250739

[53] Abarca-Zabalia J, Garcia MI, Ros AL, Marin-Jimenez I, Martinez-Gines ML, Lopez-Cauce B, Martin-Barbero ML, Salvador-Martin S, Sanjurjo-Saez M, Garcia-Dominguez JM, Fernandez LAL, Differential Expression of SMAD Genes and S1PR1 on Circulating CD4+ T Cells in Multiple Sclerosis and Crohn’s Disease. Int J Mol Sci 21 (2020). 10.3390/ijms21020676 PMC7014376 31968593

[54] Zhou L, Zhang M, Wang Y, Dorfman RG, Liu H, Yu T, Chen X, Tang D, Xu L, Yin Y, Pan Y, Zhou Q, Zhou Y, Yu C, Faecalibacterium prausnitzii Produces Butyrate to Maintain Th17/Treg Balance and to Ameliorate Colorectal Colitis by Inhibiting Histone Deacetylase 1. Inflamm Bowel Dis 24, 1926–1940 (2018). 29796620 10.1093/ibd/izy182

[55] Ehrenstein MR, Evans JG, Singh A, Moore S, Warnes G, Isenberg DA, Mauri C, Compromised function of regulatory T cells in rheumatoid arthritis and reversal by anti-TNFalpha therapy. J Exp Med 200, 277–285 (2004). 15280421 10.1084/jem.20040165 PMC2211983

[56] Lindley S, Dayan CM, Bishop A, Roep BO, Peakman M, Tree TI, Defective suppressor function in CD4(+)CD25(+) T-cells from patients with type 1 diabetes. Diabetes 54, 92–99 (2005). 15616015 10.2337/diabetes.54.1.92

[57] Vieyra-Lobato MR, Jorge VO, Laura MC, Rubén LS, Moreno-Lafont MC, Description of CD8\r ^+^\r Regulatory T Lymphocytes and Their Specific Intervention in Graft-versus-Host and Infectious Diseases, Autoimmunity, and Cancer. Journal of Immunology Research, 1–16 (2018). 10.1155/2018/3758713 PMC6098849 30155493

[58] Hsu PD, Scott DA, Weinstein JA, Ran FA, Konermann S, Agarwala V, Li Y, Fine EJ, Wu X, Shalem O, Cradick TJ, Marraffini LA, Bao G, Zhang F, DNA targeting specificity of RNA-guided Cas9 nucleases. Nat Biotechnol 31, 827–832 (2013). 23873081 10.1038/nbt.2647 PMC3969858

[59] Zhang X, Wang Y, Yuan J, Li N, Pei S, Xu J, Luo X, Mao C, Liu J, Yu T, Gan S, Zheng Q, Liang Y, Guo W, Qiu J, Constantin G, Jin J, Qin J, Xiao Y, Macrophage/microglial Ezh2 facilitates autoimmune inflammation through inhibition of Socs3. J Exp Med 215, 1365–1382 (2018). 29626115 10.1084/jem.20171417 PMC5940261

[60] Pei S, Huang M, Huang J, Zhu X, Wang H, Romano S, Deng X, Wang Y, Luo Y, Hao S, Xu J, Yu T, Zhu Q, Yuan J, Shen K, Liu Z, Hu G, Peng C, Luo Q, Wen Z, Dai D, Xiao Y, BFAR coordinates TGFβ signaling to modulate Th9-mediated cancer immunotherapy. Journal of Experimental Medicine 218, e20202144 (2021). 33914044 10.1084/jem.20202144 PMC8091105

[61] Wang Y, Fu Z, Li X, Liang Y, Pei S, Hao S, Zhu Q, Yu T, Pei Y, Yuan J, Ye J, Fu J, Xu J, Hong J, Yang R, Hou H, Huang X, Peng C, Zheng M, Xiao Y, Cytoplasmic DNA sensing by KU complex in aged CD4+ T cell potentiates T cell activation and aging-related autoimmune inflammation. Immunity 54, 632–647.e9 (2021). 33667382 10.1016/j.immuni.2021.02.003

[62] Liu H, Naismith JH, An efficient one-step site-directed deletion, insertion, single and multiple-site plasmid mutagenesis protocol. BMC Biotechnol 8, 91 (2008). 19055817 10.1186/1472-6750-8-91 PMC2629768

[63] Yu T, Gan S, Zhu Q, Dai D, Li N, Wang H, Chen X, Hou D, Wang Y, Pan Q, Xu J, Zhang X, Liu J, Pei S, Peng C, Wu P, Romano S, Mao C, Huang M, Zhu X, Shen K, Qin J, Xiao Y, Modulation of M2 macrophage polarization by the crosstalk between Stat6 and Trim24. Nat Commun 10, 4353 (2019). 31554795 10.1038/s41467-019-12384-2 PMC6761150

[64] Karim R, Teng W, Lin H, SIRT2-Mediated ACSS2 K271 Deacetylation Suppresses Lipogenesis Under Nutrient Stress. doi: 10.1101/2024.02.27.582293 (2024). PMC12058118 40331334

[65] Hou D, Yu T, Lu X, Hong JY, Yang M, Zi Y, Ho TT, Lin H, Sirt2 inhibition improves gut epithelial barrier integrity and protects mice from colitis. Proceedings of the National Academy of Sciences 121, e2319833121 (2024). 10.1073/pnas.2319833121 PMC11066986 38648480

