# Supplementary material for: SMAD2 S-palmitoylation promotes its linker region phosphorylation and TH17 cell differentiation in a mouse model of multiple sclerosis
Source: Sci Signal. Author manuscript; Available in PMC 2025 Jul 7. (PMC12234153; doi:10.1126/scisignal.adr2008)
Supplement: main supplementary [file NIHMS2087638-supplement-main_supplementary.docx]

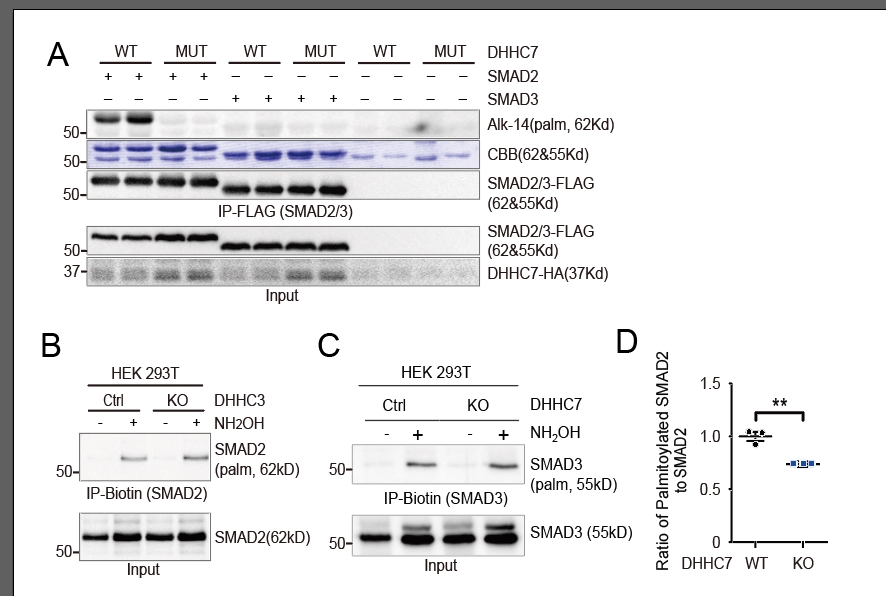


**Fig. S1. SMAD2, but not SMAD3, is palmitoylated by DHHC7.** (**A**) Analysis of the palmitoylation of SMAD2 and SMAD3 in HEK293T cells cotransfected with plasmids encoding SMAD2-FLAG or SMAD3-FLAG and with plasmid encoding HA-tagged WT DHHC7 or catalytically inactivate mutant DHHC7 (MUT-DHHC7). The indicated palmitoylated proteins were detected by Alk14 labeling and in-gel fluorescence assays. (**B**) Palmitoylation of endogenous SMAD2 in WT and DHHC3 knockout (KO) HEK293T cells was detected by acyl-biotin exchange (ABE) assay. (**C**) Palmitoylation of endogenous SMAD3 in WT and DHHC7 knockout (KO) HEK293T cells was detected by ABE assay. (**D**) Quantification of the relative amounts of palmitoylated SMAD2 in WT and DHHC7 KO T_H_17 cells from the experiments shown in Fig. 1H. The amount of palmitoylated SMAD2 was normalized to the amount of total SMAD2. Blots are representative of three experiments. Quantified data are means ± SEM. ****P* < 0.001.


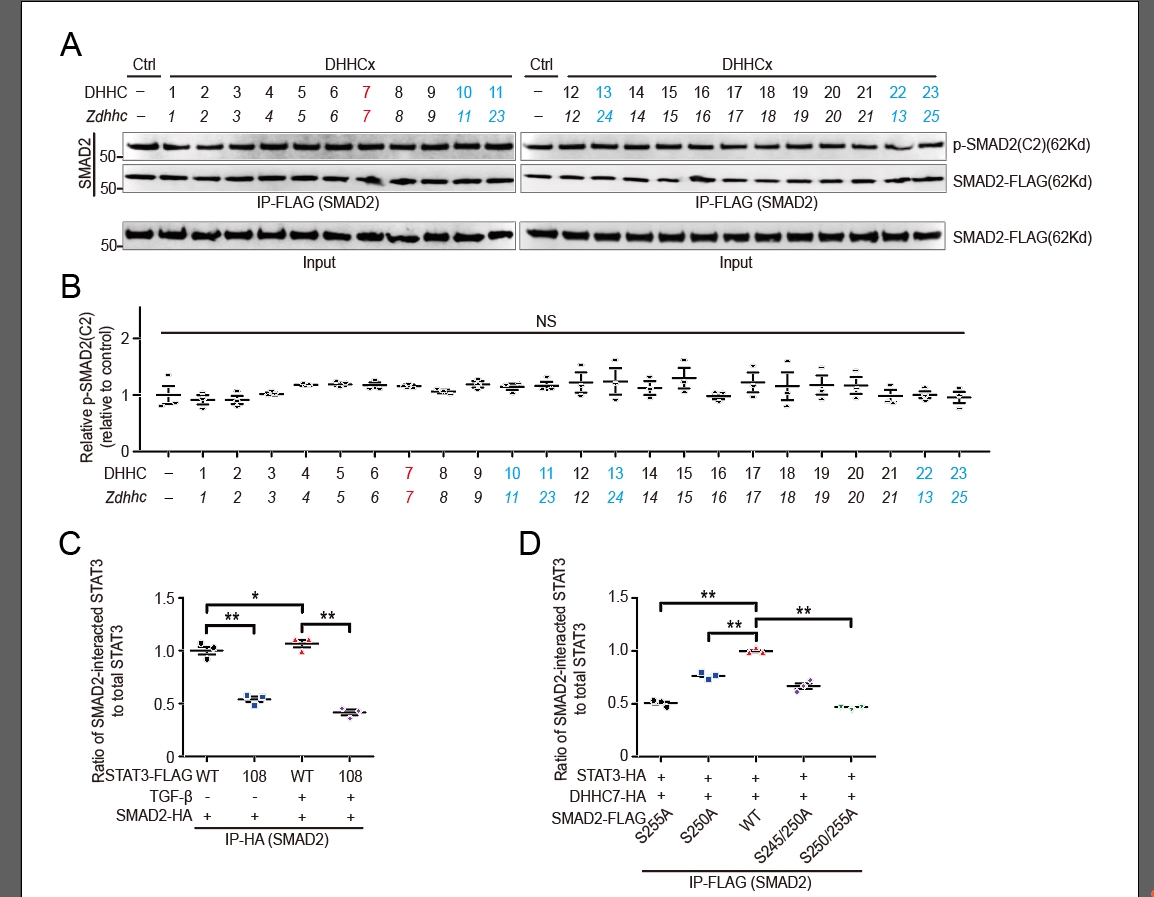


**Fig. S2.** **DHHC7-induced S-palmitoylation does not regulate the phosphorylation of SMAD2 at C-terminal residues.** (**A**) Western blotting analysis of the phosphorylation of SMAD2 at the C-terminal region [p-SMAD2(C2)] in HEK293T cells cotransfected with plasmids encoding FLAG-tagged SMAD2 and the indicated DHHCs. (**B**) The relative amounts of p-SMAD2(C2) from the experiments shown in (A) were quantified and normalized to that of total SMAD2 protein. (**C**) Quantification of the relative amounts of STAT3 bound to SMAD2 from the experiments shown in Fig. 5F. The amount of STAT3 that was pulled down by SMAD2 immunoprecipitation was normalized to the amount of total STAT3 and to the amount in the controls (WT STAT3 and SMAD2 without TGF-β), which was set at 1. (**D**) Quantification of the relative amounts of STAT3 protein that bound to SMAD2 from the experiments shown in Fig. 5I. The amount of STAT3 that was pulled down by SMAD2 immunoprecipitation was normalized to the amount of total STAT3 and to the amount in the control (WT SMAD2), which was set at 1. Quantified data are means ± SEM. ***P* < 0.01, ****P* < 0.001; NS, not significant.


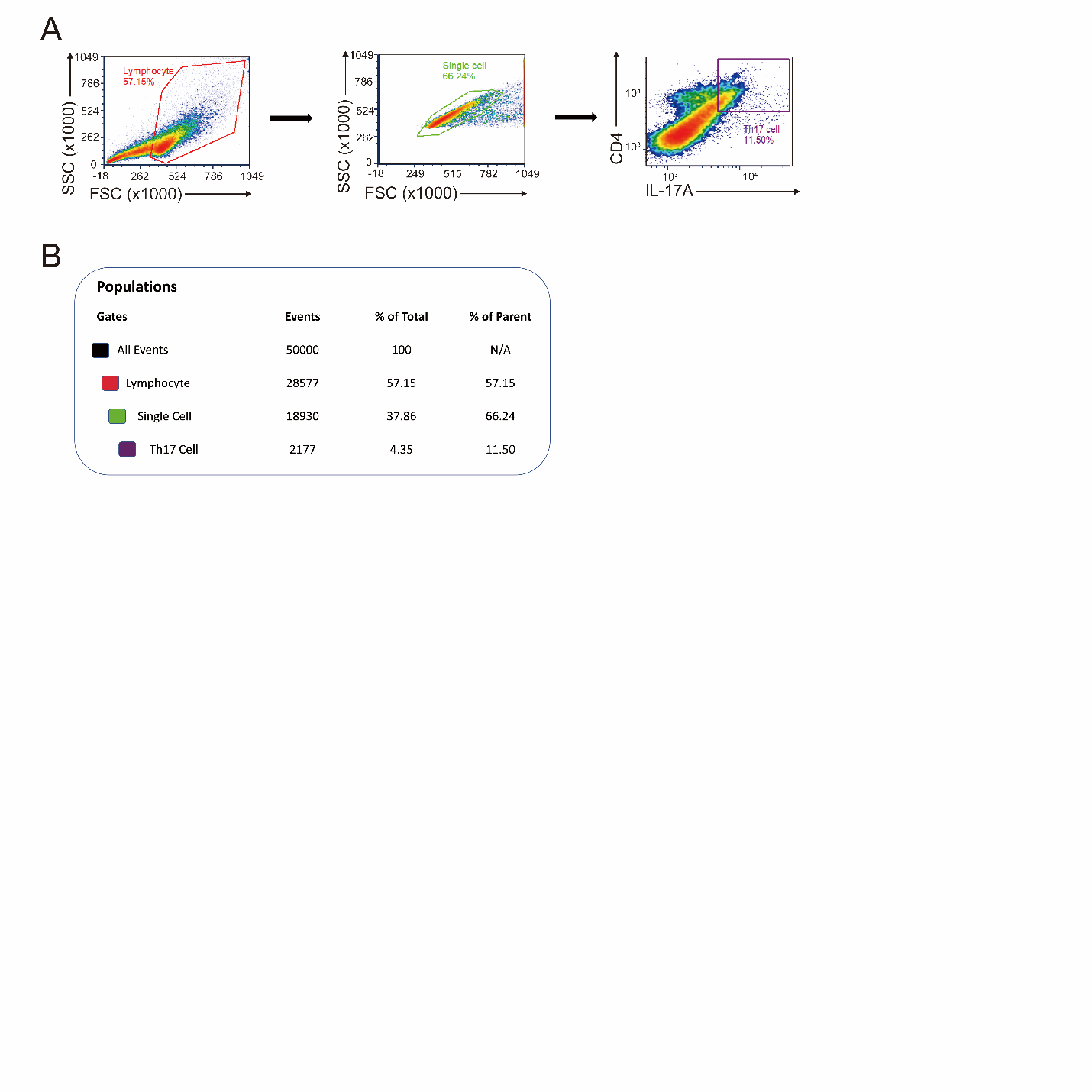


**Fig. S3. Representative flow cytometric gating strategy for T_H_17 cell analysis.** (**A**) Flow cytometry plots, showing that T_H_17 cells were gated from lymphocytes (left), single cells (middle), and IL-17A^+^CD4^+^ population (right). (**B**) Representative example for the T_H_17 cell gating strategy used in the flow cytometric analysis.


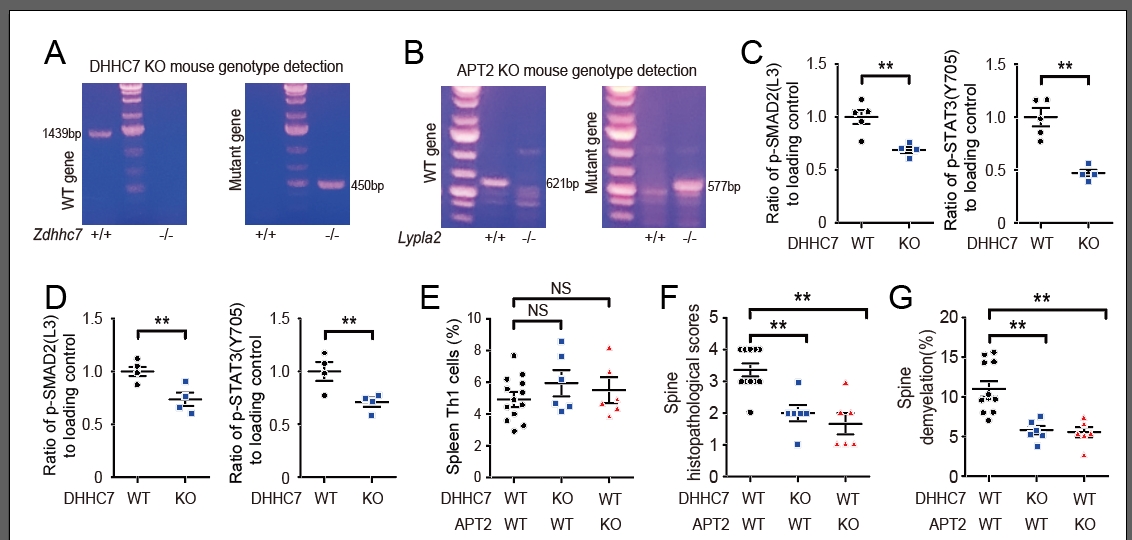


**Fig. S4. The phosphorylation of SMAD2 and STAT3 regulated by** **DHHC7 and APT2 contributes to disease severity** **in EAE mice.** (**A**) Representative genotyping results of WT and *Zdhhc7* knockout (DHHC7-KO) mice. (**B**) Representative genotyping results of WT and *Lypla2* knockout (APT2-KO) mice. The primers and methods used for mouse genotyping are found in the Materials and Methods. (**C**) The relative amounts of p-SMAD2(L3) and p-STAT3(Y705) from the experiments shown in Fig. 7C were quantified and normalized to the amount of the amounts in the loading control. Data are from four or five experiments for each group. (**D**) The relative amounts of p-SMAD2(L3) and p-STAT3(Y705) from the experiments shown in Fig. 7D were quantified and normalized to the amounts in the loading control. Data are from four experiments for each group. (**E**) Flow cytometry analysis of the T_H_1 cell populations isolated from the spleens of WT, DHHC7-KO, and APT2-KO EAE mice. The T_H_1 cells were identified as CD4^+^ and IFN-γ^+^ cells after gating on CD45^+^ cells. (**F** and **G**) H&E staining and Luxol Fast Blue staining of spinal cord sections from MOG_35-55_–immunized WT, DHHC7 KO, and APT2 KO mice from the experiments shown in Fig. 7, G and H were quantified. Immune cell infiltration into the spinal cord (F) and the severity of demyelination (G) were determined. Quantified data are means ± SEM. **P* < 0.05, ***P* < 0.01; NS, not significant.


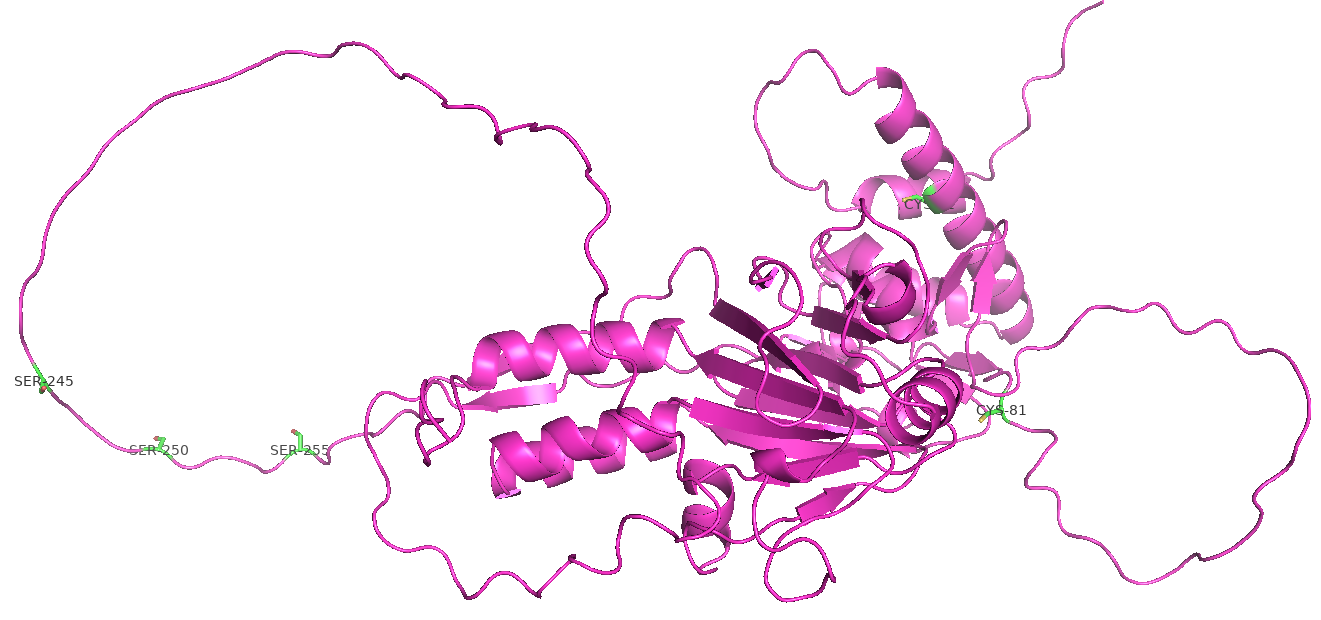


**Fig. S5. AlphaFold structure of human SMAD2 protein showing the palmitoylation site and three phosphorylation sites in the linker region.** Three serine residues (Ser^245^, Ser^250^, and Ser^255^) in the linker region that are phosphorylation sites are in an unstructured region. The SMAD2 palmitoylation site Cys^81^ is near the end of an unstructured region. All of the linker region phosphorylation sites and the palmitoylation site are accessible.


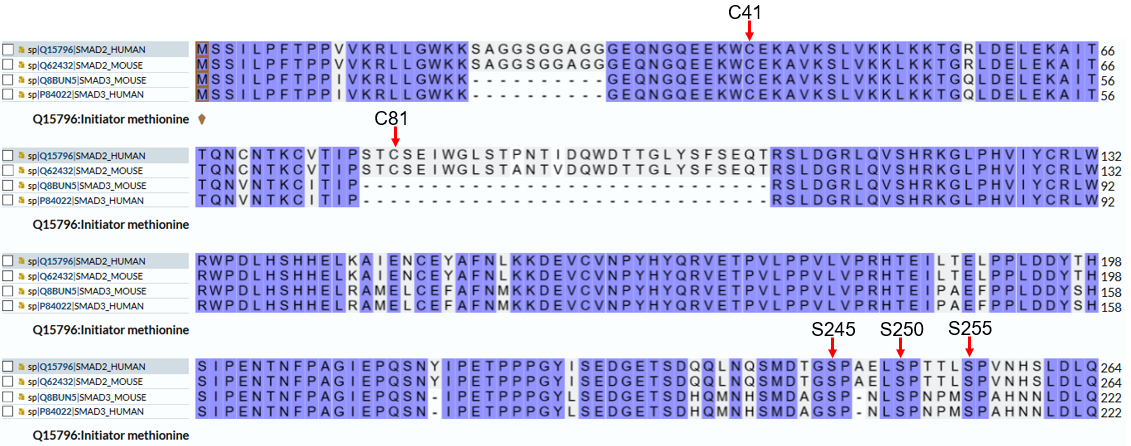


**Fig. S6. Alignment of human and mouse SMAD2 and SMAD3 protein sequences.** The main difference between SMAD2 and SMAD3 is that Cys^81^ of SMAD2 is present in an unstructured loop that is not present in SMAD3. This difference likely explains why SMAD3 is comparatively less palmitoylated than SMAD2, which in turn affects phosphorylation of sites in the linker region of SMAD3.
